# Tumor- and osteoclast-derived NRP2 in prostate cancer bone metastases

**DOI:** 10.1038/s41413-021-00136-2

**Published:** 2021-05-14

**Authors:** Navatha Shree Polavaram, Samikshan Dutta, Ridwan Islam, Arup K. Bag, Sohini Roy, David Poitz, Jeffrey Karnes, Lorenz C. Hofbauer, Manish Kohli, Brian A. Costello, Raffael Jimenez, Surinder K. Batra, Benjamin A. Teply, Michael H. Muders, Kaustubh Datta

**Affiliations:** 1 Department of Microbiology and Pathology, University of Nebraska Medical Center, Omaha, NE USA; 2 Department of Biochemistry and Molecular Biology, University of Nebraska Medical Center, Omaha, NE USA; 3 Institute for Clinical Chemistry, University Hospital Dresden, Dresden, Germany; 4 Department of Urology, Mayo Clinic, Rochester, MN USA; 5 Center for Healthy Aging and Bone Lab Dresden, Department of Medicine III, Technische Universität Dresden, Dresden, Germany; 6 School of Medicine, Division of Oncology, Huntsman Cancer Institute, Salt Lake City, UT USA; 7 Division of Anatomic Pathology, Department of Pathology and Laboratory Medicine, Mayo Clinic, Rochester, MN USA; 8 Internal Medicine, Division of Oncology & Hematology, University of Nebraska Medical Center, Omaha, NE USA; 9 Rudolf- Becker Laboratory for Prostate Cancer Research, Institute of Pathology, University of Bonn Medical Center, Bonn, Germany

**Keywords:** Bone cancer, Pathogenesis

## Abstract

Understanding the role of neuropilin 2 (NRP2) in prostate cancer cells as well as in the bone microenvironment is pivotal in the development of an effective targeted therapy for the treatment of prostate cancer bone metastasis. We observed a significant upregulation of NRP2 in prostate cancer cells metastasized to bone. Here, we report that targeting NRP2 in cancer cells can enhance taxane-based chemotherapy with a better therapeutic outcome in bone metastasis, implicating NRP2 as a promising therapeutic target. Since, osteoclasts present in the tumor microenvironment express NRP2, we have investigated the potential effect of targeting NRP2 in osteoclasts. Our results revealed NRP2 negatively regulates osteoclast differentiation and function in the presence of prostate cancer cells that promotes mixed bone lesions. Our study further delineated the molecular mechanisms by which NRP2 regulates osteoclast function. Interestingly, depletion of NRP2 in osteoclasts in vivo showed a decrease in the overall prostate tumor burden in the bone. These results therefore indicate that targeting NRP2 in prostate cancer cells as well as in the osteoclastic compartment can be beneficial in the treatment of prostate cancer bone metastasis.

## Introduction

Bone is the most common metastatic site for castration-resistant prostate cancer.^1^ Nearly 70%–80% of the patients with advanced-stage prostate cancer (PCa) develop skeletal metastases which remains as the most common cause of deaths in these patients. The 1-year survival rate in patients with metastatic PCa without incidence in bone is at nearly 87%, which is reduced to 47% in patients with bone metastasis.^2^ Only 3% of patients with bone metastasis survive after 5 years compared to 56% patients without bone metastasis.^3^ In addition, PCa patients with bone metastasis frequently suffer from skeletal-related events (SREs) such as severe bone pain, pathologic fractures, spinal cord and nerve compression syndromes, and hypercalcemia.^4,5^ Current treatment strategy for PCa patients with metastatic bone disease includes taxane-based chemotherapy, which can effectively limit the progression of the disease for a short time period but patients eventually relapse within the first year of treatment.^6–9^ To date, bone metastasis remains as a frequent and fatal complication in PCa patients, and its management is a clinical challenge. Thus, new molecular target(s) that can be therapeutically exploited are needed to improve patient outcomes. A major consideration in this effort is that treatment strategies for PCa cells metastasized to bone are also likely to influence the normal bone cells present at the site of metastatic lesions. This is because the metastatic disease results from an interplay between cancer cells and the bone cells namely osteoblasts and osteoclasts, which contributes to a vicious cycle that provides a fertile soil for metastatic growth of cancer cells in the bone.^1,10,11^ Radiographic studies of PCa patients with bone involvement characterized bone metastases as osteoblastic lesions as opposed to osteolytic lesions with decreased bone mineral density.^12^ In fact, PCa bone metastases show a heterogeneous blend of osteoblastic and osteolytic functions with the balance shifted to favor osteoblastic metastasis. Because of this multifaceted interplay between PCa cells and bone cells, it is important to test how a potential therapy against metastatic PCa cells can influence the function of bone cells.

The NRPs are type-I transmembrane glycoproteins belonging to a family of non-tyrosine kinase, cell surface receptors that function in multiple cellular signaling pathways in normal physiology as well as pathological conditions.^13,14^ They are over-expressed in various neoplasms and correlates with stress-induced cancer survival, progression, metastasis, and poor prognosis.^15–17^ We have previously observed the role of NRP2 in protecting PCa cells against chemotherapies in vitro.^18,19^ In this study, we addressed how inhibition of the functions of NRP2 affects PCa cells in PCa bone metastasis, thus highlighting the potential of NRP2-targeted therapy to benefit PCa patients with bone metastasis. Our study also investigated the function of NRP2 in osteoclasts present in the bone microenvironment in promoting PCa bone metastasis, as it is known to be expressed in these cells.^20^ We have elucidated a novel function of NRP2 as a negative regulator of osteoclast differentiation and function in PCa bone metastasis. Interestingly, our findings imply that PCa-induced NRP2 expression in osteoclasts is necessary for low osteolytic activity in PCa bone metastasis with mixed lesions and that PCa, which is predominantly osteolytic in nature, evades NRP2 inhibition. Interestingly, knocking out NRP2 in osteoclasts in a PCa model of bone metastasis significantly inhibited tumor growth. Our study thus eludicated the overall effect of targeting NRP2 in both cancer cells and osteoclasts in PCa bone metastasis, which can prove to be beneficial in the development of an effective treatment strategy.

## Results

### NRP2 is a potential therapeutic target in treating PCa bone metastasis

#### High expression of NRP2 in human PCa bone metastasis

**Fig. 1:**
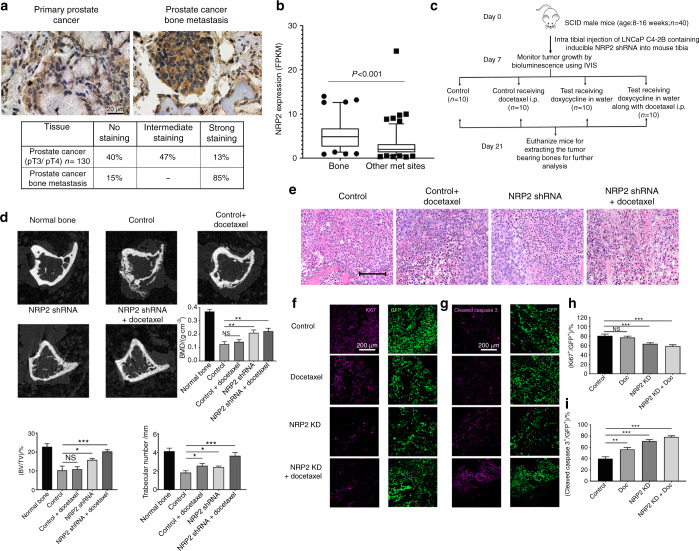
Targeting NRP2 in prostate cancer cells in PCa bone metastasis is effective in combination with chemotherapy. **a** Top: Representative immunohistochemical staining of NRP2 expression in human primary prostate cancer and prostate cancer bone metastasis. Bottom: Table showing the percentage positivity of NRP2 staining intensity distinguished as No, intermediate and strong staining in human primary prostate cancer and prostate cancer bone metastasis tissues. **b** Dot-plot graph depicting the expression of NRP2 from RNA- seq data of mCRPC patients comparing between metastatic sites: bone vs other soft or visceral tissue sites. **c** Schematic diagram of the intratibial injection of LNCaP C4-2B containing inducible NRP2 shRNA. At day 7, the mice were sorted based on the bioluminescence imaging and divided into four groups: control, docetaxel alone, doxycycline in water and combination of doxycycline in water and docetaxel. **d** Representative micro-CT images of trabecular compartment of the proximal tibia for normal bone and all four treatment groups. Graphs showing bone mass density (BMD), bone volume/tissue volume (BV/TV) and trabecular number per mm area for each group. For each group, *n* = 6 mice were used to analyze tumor bearing bones from two independent experiments. **e** Representative H&E images for each treatment group depicting the status of tumor cells in the tibia of mice. **f**, **g** Immunofluorescence images showing the expression of Ki67 (proliferation marker) and cleaved caspase 3 (apoptosis marker) for each group respectively in pink along with respective area showing tumor cells in green. **h**, **i** Graphs representing the quantification of the mean corrected total cell fluorescence for Ki67 and cleaved caspase 3 (pink) with respect to tumor cells (green). All data are shown as mean ± standard error of mean (SEM). Statistical significance was calculated by student *t*-test and *P* value denoted as NS (not significant), * (0.05), ** (0.01), *** (0.001)

To address whether NRP2 is present in human PCa bone metastasis, we evaluated its expression by immunohistochemistry both in human primary PCa tissues and in PCa bone metastasis. For this, a bone metastatic tissue microarray was purchased from TriStar Technology (Rockville, MD, USA.) which contained samples of fifty patients. The primary PCa cohort was obtained from radical prostatectomy specimens of 130 patients with non-metastasized but locally advanced PCa (pT3a or pT3b/4, all pN0) treated at Mayo Clinic between 1992 and 1997 (refer to the methods section). On comparing the tissues, heterogeneous expression of NRP2 was observed in primary PCa while expression of NRP2 was higher and more homogenous in PCa bone metastasis (Fig. 1a). Nearly 85% of the tissues from PCa bone metastasis showed high NRP2 expression. We also observed no expression of NRP2 in the normal prostate (Supplementary Fig. 1a). Analysis of RNA-seq data from metastatic castration-resistant PCa (CRPC) patients revealed that NRP2 expression in bone is significantly higher as compared to the other visceral and soft tissue metastatic sites (https://www.cbioportal.org/study/summary?id=prad_su2c_2019) (Fig. 1b).^21^ These results therefore indicated a potential role of NRP-2 in bone metastatic PCa.

#### Targeting NRP2 in combination with chemotherapeutic agents is effective against PCa cells in a bone metastasis model

Our previous in vitro studies implicated NRP2 in promoting survival of cancer cells under chemotherapeutic stress via autophagy.^18,19,22^ Since we observed increased expression of NRP2 in PCa metastasized to bone, we hypothesized that targeting the NRP2 axis in PCa bone metastasis will sensitize cancer cells to chemotherapy. We therefore tested whether depletion of NRP2 in the mouse model of bone metastasis would sensitize prostate tumor to docetaxel. We have developed stable shNRP2 expressing clones of LNCaP C4-2B (an mCRPC cell line), where shRNA can be inducibly expressed upon administration of doxycycline. The requirement of NRP2 for PCa growth in bone was tested by implanting shNRP2-expressing LNCaP C4-2B cells in the tibia of immunocompromised mice and then knocking down NRP2 from cancer cells in combination with docetaxel (Fig. 1c). Depletion of NRP2 in tumor cells was confirmed by mRNA analysis of tumor cells isolated from the mouse bones (Supplementary Fig. 1b). No change was observed in the weight of the mice as well as their ability to drink water containing either sucrose alone or doxycycline in sucrose in all the experimental groups (Supplementary Fig. 1c, d). LNCaP C4-2B develops bone metastases that is characterized by their loss/destruction/reformation of bone tissue measured by bone morphometric analysis. Inhibition of bone metastases formation is consequently indicated by an increase in bone mass. Bone morphometric analysis showed a significant bone loss in the control (LNCaP C4-2B only) as well as in docetaxel only group. By contrast, NRP2 depletion in the tumor cells reduced bone destruction, indicating an inhibition of growth of PCa cells in bone. The bone mass recovery was more profound in the combination group which had NRP2 depletion in tumor cells and received docetaxel (Fig. 1d). Evaluation of standard H&E-stained and EDTA-decalcified slides of the bone metastatic site revealed intact tumor cells without any necrosis in the control group. Docetaxel treatment alone clearly increased the necrotic area. Also, the group with silenced NRP2 in the tumor cells demonstrated increased necrosis. Almost all the metastatic lesions became necrotic when combining NRP2 depletion with taxane-based chemotherapy (Fig. 1e). To validate these results, we measured Ki67 proliferation as well as cleaved caspase 3 as an indicator of apoptosis in the metastatic lesions. In comparison to the control, apoptosis was increased in the groups receiving chemotherapy alone and NRP2 depletion alone. Metastatic lesions treated with both NRP2 silencing and docetaxel showed the highest proportion of apoptotic cells. In line with these results, the fraction of proliferating tumor cells was significantly decreased in both the NRP2-depletion and the combination group compared with the control (Fig. 1f–i). These data cumulatively suggest that targeting NRP2 in PCa cells growing in bone may enhance therapy in combination with standard chemotherapeutic agents.

### Targeting the NRP2 axis affects the function of osteoclasts differentiated by factors released by the metastatic PCa

While the above results suggested NRP2 is a potential target to treat PCa patients with bone metastasis, the potential effect of targeting NRP2 in non-cancer cells in the tumor microenvironment is not known. Osteoclasts express NRP2 and are reported to be early propagators of PCa bone metastasis. Thus, we investigated how inhibition of NRP2 might affect the function of these cells in the context of PCa bone metastasis.

#### PCa cell induced NRP2 expression in osteoclasts

We used two bone metastatic PCa cell lines: LNCaP C4-2B and PC3. Being a derivative of LNCaP with its ability to metastasize to bone, LNCaP C4-2B is a PCa cell line that can induce bone lesions with mixed osteoblastic and osteolytic function.^23,24^ PC3 is a bone metastatic PCa cell line, which promotes high osteolytic lesions with minimal or no osteoblastic function.^25,26^ Osteoclast precursors were isolated from the bone marrow of C57BL6 mice (forelimbs and hind limbs) and were differentiated using RANKL and M-CSF (control condition) and conditioned medium (CM) from PC3 and LNCaP C4-2B cells separately. M-CSF and RANKL signaling is central to osteoclast differentiation under physiological condition.^27^ The differentiation was confirmed by tartarate-resistant acid phosphatase (TRAP) staining, released TRAP levels in the media and a pit resorption assay (Supplementary Fig. 2a–f, h). The expression of osteoclastic genes such as RANK, TRAP, cathepsin-k, DC-stamp, ATP6V0D2, metalloprotease 9 and carbonic anhydrase (Supplementary Fig. 2g) was also measured as markers for differentiation.

**Fig. 2:**
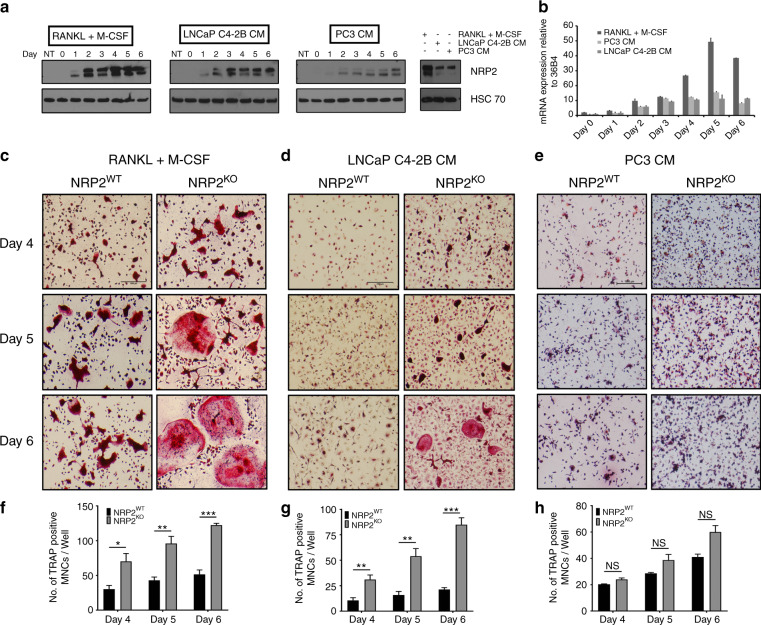
NRP2 ablation increases osteoclast differentiation. **a** Protein analysis of NRP2 expression in a time course (0-6 days) in osteoclasts differentiated under different conditions in comparison to osteoclastic precursors under the conditions of RANKL (100 ng·mL^−1^) and M-CSF (20 ng·mL^−1^), LNCaP C4-2B CM and PC3 CM. Right: Comparison of NRP2 protein levels in RANKL and M-CSF, LNCaP C4-2B CM and PC3 CM at day 6. **b** Graph showing the expression of NRP2 at mRNA level in all the three conditions in the time course of osteoclast differentiation. Knockout of NRP2 in osteoclastic precursors isolated from NRP2 Fl/Fl; CSF1R-Cre transgenic mice by addition of 4-HydroxyTamoxifen in vitro and differentiated into osteoclasts in the presence of RANKL and M-CSF, LNCaP C4-2B CM and PC3 CM. **c**–**e** TRAP staining showing osteoclast differentiation at day 4–6 after depletion of NRP2 in RANKL and M-CSF, LNCaP C4-2B CM and PC3 CM, respectively. **f**–**h** Quantification of the TRAP positive multinucleated cells per well and comparison between NRP2WT and NRP2KO osteoclasts represented as a graph in RANKL and M-CSF, LNCaP C4-2B CM and PC3 CM, respectively. All values reported as mean± SEM from three independent experiments. Statistically significant *P* value denoted as * (0.05), ** (0.01), *** (0.001)

We tested for the expression of NRP2 and found that osteoclasts expressed NRP2 in all three conditions. A time course study (0–6 days) in the differentiating osteoclasts revealed that NRP2 expression increases with time both at the transcriptional and protein levels (Fig. 2a, b).

#### NRP2 differentially regulates the function of osteoclasts differentiated by osteosclerotic vs osteolytic PCa factors

**Fig. 3:**
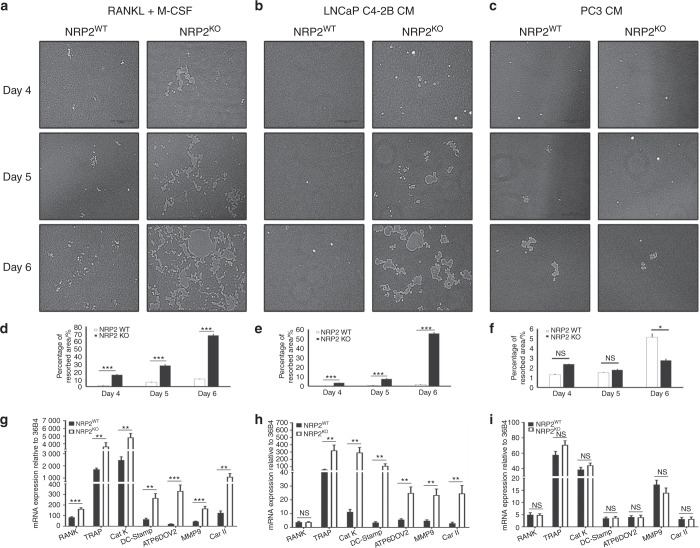
Depletion of NRP2 escalates the resorptive function and gene expression in osteoclasts. Knockout of NRP2 in osteoclastic precursors isolated from NRP2 Fl/Fl; CSF1R-Cre transgenic mice by addition of 4-HydroxyTamoxifen in vitro and differentiated into osteoclasts in the presence of RANKL and M-CSF, LNCaP C4-2B CM and PC3 CM on a 24-well osteoassay plate. **a**–**c** Representative images of pit resorption to compare the resorbed area in NRP2WT and NRP2KO osteoclasts in RANKL+M-CSF, LNCAP C4-2B CM and PC3 CM respectively. **d**–**f** Quantification and comparison of the percentage resorbed area in NRP2WT and NRP2KO osteoclasts in RANKL and M-CSF, LNCaP C4-2B CM and PC3 CM respectively. **g**–**i** Graphical representation of the osteoclastic gene expression at mRNA in NRP2WT and NRP2KO condition treated respectively with RANKL and M-CSF, LNCaP C4-2B CM and PC3 CM. All values reported as mean± SEM from three independent experiments and represented as a bar graph with error bar. Statistically significant *P* value denoted as * (0.05), ** (0.01), *** (0.001)

To understand the significance of inhibiting NRP2 in osteoclasts in bone metastatic PCa, we depleted NRP2 in osteoclast precursors and induced osteoclastic differentiation using control and PCa CM treatment. NRP2 was depleted/deleted either by silencing with siRNA against NRP2 or using a transgenic mouse model, NRP2 ^*Flox/Flox*^; CSF1R-cre where NRP2 is specifically deleted in osteoclast precursors by the addition of hydroxytamoxifen. In the RANKL and M-CSF condition, we found extremely large multinucleated structures in NRP2-depleted or -deleted osteoclasts by TRAP staining and a significant increase in TRAP activity (Fig. 2c, f, Supplementary Figs. 3a, 4a, 4e). We also observed a greater number of osteoclasts in the NRP2-deleted condition (Fig. 3c). Similarly, depletion of NRP2 in osteoclasts exposed to LNCaP C4-2B CM resulted in elevated osteoclast differentiation and function compared with NRP2-intact cells (Fig. 2d, g). These cells also fused into giant, multinucleated structures with increased TRAP activity, even by day 4 (Fig. 2d, g, Supplementary Figs. 3c, 4b, g).

Interestingly, in PC3 CM-induced osteoclasts, absence of NRP2 did not affect osteolytic differentiation and activation. Further, osteoclasts induced by PC3 CM showed no difference in TRAP staining and TRAP activity after NRP2 reduction compared to the control (Fig. 2e, h, Supplementary Figs. 3e, 4c, i). The number of osteoclasts attached to the plate increased after NRP2 deletion in PC3 CM, although the increase did not reach significance (Fig. 2h). We were able to efficiently delete or deplete NRP2 by knockout or siRNA in all three conditions (Supplementary Figs. 3b, d, f and 4d, f, h).

To validate the augmented osteoclast activity after NRP2 depletion, a pit resorption assay was performed. The results demonstrated increased bone resorption in differentiated NRP2-null osteoclasts in comparison to NRP2-positive osteoclasts exposed to RANKL and M-CSF (Fig. 3a, d). On day 4 of differentiation, NRP2-depleted osteoclasts started to fuse with each other and form a polykaryon. By day 5, these multinucleated osteoclasts started resorbing the surface of the osteoassay plate actively. We observed a significant increase in the area of the resorbed pit in NRP2-deleted osteoclasts in comparison to NRP2-expressing cells (Fig. 3a, d). By day 6, nearly 70% of the surface of the osteoassay plate was resorbed due to deletion of NRP2. Thus, the pits formed by NRP2-depleted osteoclasts were larger when compared to those from NRP2-positive osteoclasts (Fig. 3a).

A striking difference was also observed in the expression of osteoclastic markers. In comparison to the control osteoclasts, NRP2-depleted osteoclasts expressed significantly higher osteoclast markers. RANK expression increased by 10-fold in NRP2-knockout osteoclasts compared to NRP2-expressing cells. The expression of TRAP, cathepsin K DC-STAMP, ATP6V0D2, carbonic anhydrase II (Car II), ATP6i and matrix metalloprotease 9 (MMP9) increased in the absence of NRP2 in osteoclasts (Fig. 3g, Supplementary Fig. 4j).

Like RANKL and M-CSF treatment, LNCaP C4-2B CM treatment led to increased resorptive function of NRP2-deleted osteoclasts. NRP2 ablation increased pit absorption by nearly 45% by day 6 (Fig. 3b, e). Further, osteoclasts in LNCaP C4-2B CM expressed exponentially higher levels of osteoclast-associated genes following NRP2 deletion compared to the control (Fig. 3h, Supplementary Fig. 4k). However, resorption function of the NRP2-downregulated osteoclasts in PC3 CM did not differ from their control counterparts (Fig. 3c, f). Furthermore, deletion of NRP2 caused either no change or a decrease in the expression of osteoclastic genes in osteoclasts in PC3 CM (Fig. 3i, Supplementary Fig. 4l). Together, the data suggested that NRP2 blockade increases osteoclastogenesis in osteoclasts induced by osteosclerotic LNCaP C4-2B CM, but not in osteoclasts induced by osteolytic PC3 CM.

### NRP2 regulates downstream signaling in osteoclasts under different differentiation stimuli

#### NRP2 regulates the expression and function of NFATC1 in osteoclasts treated with RANKL and M-CSF, and LNCaP C4-2B CM but not PC3 CM

**Fig. 4:**
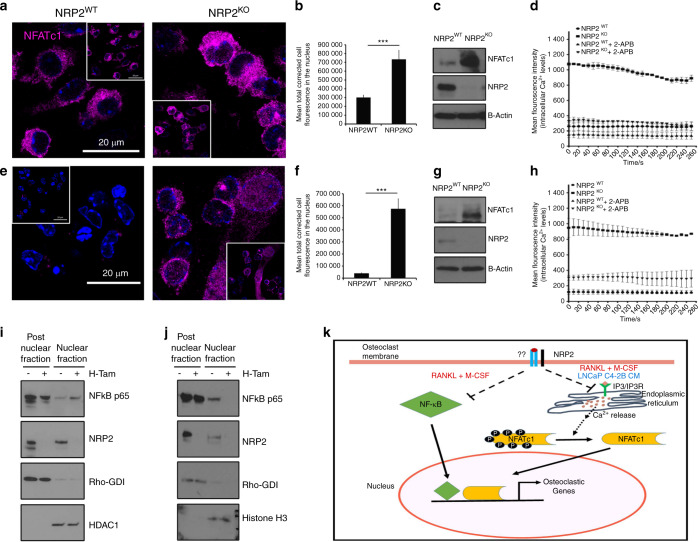
NFATc1 is regulated by NRP2. NRP2 depletion in osteoclastic precursors isolated from NRP2 Fl/Fl; CSF1R-Cre transgenic mice by addition of 4-HydroxyTamoxifen in vitro and differentiated into osteoclasts under different conditions. Confocal images showing NFATc1 translocation into the nucleus compared between NRP2WT and NRP2KO osteoclasts at day 3 of osteoclastic differentiation in **a** RANKL and M-CSF, **e** LNCaP C4-2B CM. Insert represents the total field from which the magnified image was taken. **b, f** Quantification of the mean corrected total cell fluorescence between NRP2WT and NRP2KO osteoclasts in RANKL and M-CSF and LNCaP C4-2B CM respectively. All values reported as mean± SEM from three independent experiments and represented as a bar graph with error bar. The statistical significance, *P* value depicted as *(<0.05),**(<0.01), ***(<0.001). **c**, **g** Western blot showing the total NFATc1 protein in NRP2WT and NRP2KO osteoclasts at day 3 in RANKL and M-CSF as well as LNCaP C4-2B CM respectively. Mean Fluorescence Intensity proportional to the levels of intracellular Ca^2+^ in NRP2WT and NRP2KO osteoclasts at day 3 of osteoclastic differentiation with the addition of 2-APB, an allosteric inhibitor of IP3-induced Ca^2+^ release in **d** RANKL and M-CSF, **h** LNCaP C4-2B CM. Western blot analysis of NF-κB translocation status in nuclear and post nuclear fractions of protein lysates isolated from osteoclasts depleted of NRP2 under the conditions of **i** RANKL and M-CSFand **j** LNCaP C4-2B CM. Rho-GDI and HDAC1 used as loading controls for post-nuclear and nuclear proteins, respectively. **k** Schematic illustration of the molecular pathways through which NRP2 regulates the gene transcription in osteoclasts. In RANKL and M-CSF, NRP2 inhibits NFATc1 and NF-κB translocation into the nucleus while only NFATc1 nuclear translocation is blocked by NRP2 in LNCaP C4-2B CM via the regulation of Ca^2+^ release from endoplasmic reticulum

Our results indicated that NRP2 regulates the expression of osteoclastic genes in osteoclasts treated with RANKL and M-CSF as well as LNCaP C4-2B CM. We therefore evaluated whether NRP2 controls NFATc1, a key transcription factor in osteoclastogenesis, under these conditions.^28^ Immunofluorescence studies showed that NFATc1 localization in the nucleus (a measure of its activity) was higher in NRP2-null osteoclasts than in NRP2-expressing osteoclasts during early differentiation in day 2 and day 3 following RANKL and M-CSF, and LNCaP C4-2B CM treatments (Fig. 4a, b, e, f Supplementary Fig. 5a–c, e). NFATc1 expression continued to increase (Fig. 4e). The control cells showed no or less localization of NFATc1 in the nucleus, suggesting NFATc1 is mainly in the cytoplasm of osteoclasts treated with LNCaP C4-2B CM (Fig. 4e). Since NFATc1 functions as its own transcription factor and thus increases its own expression, we analyzed the total level of NFATc1 in osteoclasts following NRP2 knockout. Osteoclasts with NRP2 knockout treated with either RANKL and M-CSF or LNCaP C4-2B CM expressed higher levels of NFATc1 than osteoclasts with NRP2 (Fig. 4c, g, Supplementary Fig. 5d). Our results therefore suggested that NRP2 negatively regulates NFATc1 in differentiating osteoclasts stimulated with LNCaP C4-2B CM or by RANKL and M-CSF (Fig. 4k).

In contrast to these findings, CM from the osteolytic PCa cells, PC3, induced low or no activation of NFATc1 in osteoclasts even after deletion of NRP2. NFATc1 localized in the cytoplasm but not the nucleus in both NRP-2 expressing as well as depleted osteoclasts in PC3 CM, as seen by immunofluorescence staining (Supplementary Fig. 6a, b). We also found very low or no expression of total NFATc1 in the PC3 CM-treated osteoclasts, and no change in the total protein was observed after depletion of NRP2 (data not shown). This suggests that NRP2 does not regulate NFATc1 in PC3 CM-induced osteoclasts.

#### NRP2 regulates intracellular calcium (Ca^2+^) levels upstream of NFATC1 in osteoclasts

The activation of NFATc1 involves the upstream function of the Ca^2+^/calmodulin pathway. Transient increases in the levels of intracellular Ca^2+^ activate calmodulin, leading to activation of its effector proteins, the calcium/calmodulin-activated kinases (CaMKs) and calcineurin. The active calcineurin dephosphorylates NFATc1 in a Ca^2+^-dependent manner to promote its translocation from the cytoplasm to the nucleus for transcription of osteoclastic genes.^29^ To understand how NFATc1 is activated in osteoclasts in the absence of NRP2, we monitored the levels of intracellular Ca^2+^ using Fluo-4 in osteoclasts in the presence and absence of NRP2. For this, we used calcium indicator, Fluo-4, which binds to intracellular Ca^2+^ and monitor the changes in the levels of intracellular Ca^2+^. In RANKL and M-CSF-treated osteoclasts, NRP2 depletion led to a 3-fold increase in intracellular Ca^2+^ levels compared to NRP2-expressing osteoclasts (Fig. 4d). Interestingly, intracellular Ca^2+^ levels were low in LNCaP C4-2B CM-treated osteoclasts until removal of NRP2, which caused a fourfold increase (Fig. 4h). In osteoclasts, binding of inositol-1, 4, 5-trisphosphate (IP3) to its receptor IP3R can release Ca^2+^ from the endoplasmic reticulum stores.^30^ To address whether IP3/ IP3R signaling causes this burst of intracellular Ca^2+^ from the ER, we incubated osteoclasts with 2-APB, an allosteric inhibitor of IP3-induced Ca^2+^ release.^31^ Treatment with 2-APB decreased intracellular Ca^2+^ levels in osteoclasts treated with RANKL and M-CSF (Fig. 4d). This decrease was more profound in NRP2-knockout osteoclasts. Treatment with 2-APB did not change the intracellular levels of Ca^2+^ in LNCaP C4-2B CM treated-osteoclasts, but a significant decrease in Ca^2+^ levels was observed in NRP2-depleted osteoclasts (Fig. 4h). These data together imply that increased intracellular Ca^2+^ levels mediate the hyper activation of NFATc1 in the absence of NRP2 in osteoblasts stimulated with RANKL and M-CSF as well as LNCaP C4-2B CM. Further, this increase in intracellular Ca^2+^ is due to the activation of IP3/IP3R signaling, which causes the calcium channels to release Ca^2+^ from the ER reserves.

#### Osteoclastic NF-κB is not controlled by NRP2 in bone metastatic prostate cancer

The coordinated function of NFATc1 with other factors such as NF-κB has been widely studied in osteoclastogenesis. Hence, we investigated whether NRP2 regulates osteoclast function via NF-κB. For this, we depleted NRP2 in osteoclastic precursors and analyzed NF-κB protein localization via fractionation of nuclear and post-nuclear proteins (membrane and cytoplasmic proteins). Control and NRP2-depleted cells treated with RANKL and M-CSF expressed high levels of NF-κB. NRP2-depleted osteoclasts also exhibited increased nuclear expression of NF-κB compared with controls, which was further increased with RANKL and M-CSF stimulation (Fig. 4i). These data were confirmed by immunofluorescence staining of NF-κB (Supplementary Fig. 7a, b). In summary, RANKL/M-CSF-treated osteoclasts express NRP2, which controls the expression and localization of NFATc1 and NF-κB during osteoclastic gene transcription. The absence of NRP2 in osteoclasts leads to their hyperactivation because of increased activity of NFATc1 and NF-κB. Next, we evaluated the status of NF-κB after depletion of NRP2 in osteoclasts treated with LNCaP C4-2B CM. NRP2-expressing osteoclasts expressed NF-κB in the nucleus; however, removal of NRP2 decreased these levels (Fig. 4j). This data contradicts our findings in RANKL and M-CSF-induced osteoclasts, suggesting that the increase in osteoclastic activity following NRP2 depletion in osteoclasts differentiated by LNCaP C4-2B CM is not dependent on NF-κB, but rather works centrally through NFATc1. In the presence of PC3 CM, no change in NF-κB was observed between control and NRP2-ablated osteoclasts. NF-κB was localized in the cytosolic compartment but completely absent in the nucleus in NRP2-expressing and -knockout osteoclasts (Supplementary Fig. 6c). These data indicate that NF-κB is rendered inactive in both NRP2-expressing or knockout conditions in PC3 CM-treated osteoclasts.

Altogether, NFATc1 is central to osteoclastogenesis induced by metastatic PCa cells, which exhibit mixed osteoblastic/osteolytic lesions. NRP2 controls NFATc1 to control osteoclast differentiation and function. However, PC3 CM-treated osteoclasts are not dependent on either NFATc1 or NF-κB for osteoclastogenesis and evade the regulation of NRP2.

### Osteoclastogenesis induced by PC3 factors follows a unique differentiation program that bypasses NRP2

Our observations in the TRAP staining of osteoclasts induced by CM of PC3 cells suggest that these osteoclasts do not differentiate into large multinucleated cells but are restricted to either 2 or 3 nucleated osteoclasts (Fig. 2e). Also, the pits formed by these osteoclasts are small and high in number (Fig. 3c, f). This indicates that CM induces osteoclast to an extent that they are active even in 2–3 nucleated state with moderate expression of osteoclastic genes sufficient for the resorptive function.

#### PC3 CM induced-osteoclastogenesis is independent of RANKL

**Fig. 5:**
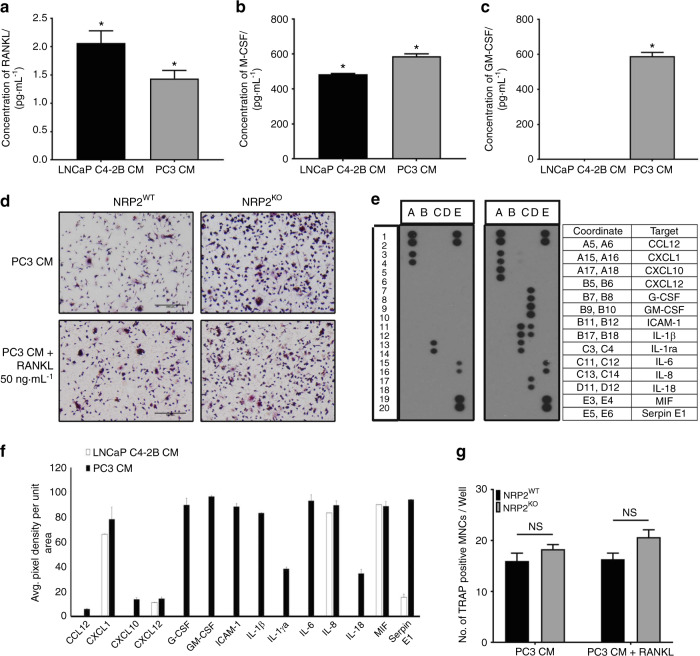
Cytokine analysis of CM collected from LNCaP C4-2B and PC3. Graphical representation of absolute **a** RANKL, **b** M-CSF and **c** GM-CSF concentration in pg·mL^−1^ in LNCaP C4-2B and PC3 CM by ELISA. **d** Osteoclastic precursors in NRP2WT and NRP2KO condition treated with PC3 CM without and with RANKL at 50 ng·mL^−1^ and graphically represented in **g**. **e** Human cytokine array membrane containing 36 different cytokines were incubated with CM from LNCaP C4-2B and PC3 mixed with biotynylated detection antibodies. The bound cytokine developed with Streptavidin-HRP and chemiluminescent detection reagents. Light produced at each spot is proportional to the amount of cytokine bound. Dot blot showing the presence of cytokine in LNCaP C4-2B CM (left) and PC3 CM (right) along with the table showing the complete list of cytokines that were found in the CM. **f** Average pixel density of each cytokine observed in the dot blots was analyzed by Imagej software and graphically represented to compare the cytokines obtained in LNCaP C4-2B and PC3 CM

PC3 and LNCaP C4-2B cells secrete different factors to regulate the functioning of osteoclasts. Hence, we evaluated the secreted factors in the CM of PC3 and LNCaP C4-2B cells. M-CSF, which is a determinant in the commitment of myeloid progenitors into osteoclast and macrophage precursors, is also involved in osteoclastic differentiation via the RANKL signaling.^27^ We analyzed these molecules by ELISA and detected very low levels of RANKL, nearly 1 pg·mL^−1^ and 2 pg·mL^−1^ in the CM of PC3 and LNCaP C4-2B cells, respectively (Fig. 5a); M-CSF levels were around 500 pg·mL^−1^ in LNCaP C4-2B CM and 600 pg·mL^−1^ in PC3 CM (Fig. 5b). The presence of M-CSF in the CM of PCa cell lines indicates early commitment of myeloid progenitors to osteoclastic precursors, as seen by TRAP staining where we observed mononucleated TRAP-positive cells. In a metastatic bone microenvironment, RANKL could be provided by other cells such as osteoblasts. Hence, we tested the differentiation and function of osteoclasts in PC3 CM supplemented with RANKL at a concentration of 50 ng·mL^−1^ (a dose frequently used by researchers to induce osteoclast differentiation). We observed that even at day 6 of differentiation, the osteoclasts looked similar with and without RANKL. Further, no change was observed after depletion of NRP2 in these conditions (Fig. 5d, g). This suggests that the RANKL/RANK pathway is not central to the differentiation of osteoclasts in the presence of PC3 CM.

#### GMCSF in the CM of PC3 cells promotes differentiation toward an immature dendritic cell phenotype and thus uncouples the negative regulation of NRP2

To evaluate the osteoclast-promoting factors in the CM of PCa cell lines, we conducted a cytokine array of 36 different cytokines. Interestingly, LNCaP C4-2B CM contained only a few cytokines, such as CXCL1, CXCL12, IL-8, MIF, and Serpin E1 in. Of these, IL-8 and CXCL1, which are positive inducers of osteoclasts, were present in high levels (Fig. 5e, f). On the contrary in PC3 CM, we detected several known osteoclast-inducing factors such as CCL12, CXCL1, CXCL10, CXCL12, G-CSF, GM-CSF, IL-1β, IL-1F3, IL-6, IL-8, IL-18, MIF, and Serpin E1 (Fig. 5e, f).^32–40^

**Fig. 6:**
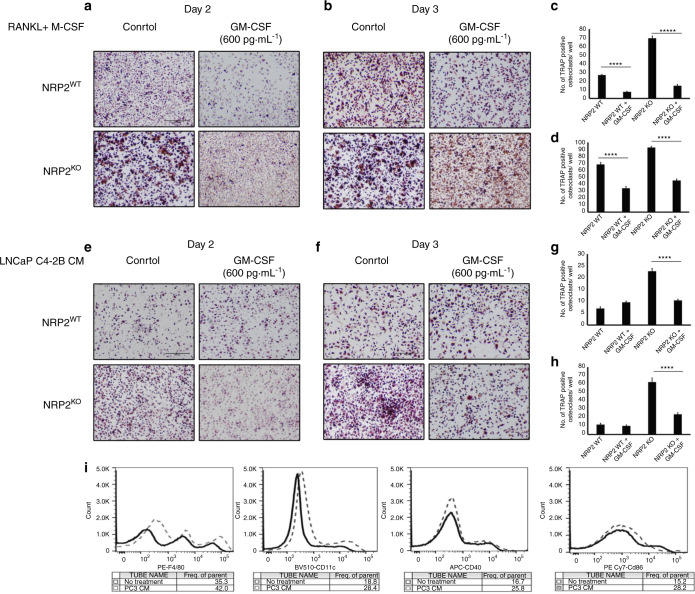
Addition of GM-CSF blocks the differentiation and fusion of osteoclasts. Knockout of NRP2 in osteoclastic precursors isolated from NRP2 Fl/Fl; CSF1R-Cre transgenic mice by addition of 4-HydroxyTamoxifen in vitro and differentiated into osteoclasts in the presence of RANKL and M-CSF, LNCaP C4-2B CM and PC3 CM. Under these conditions, one set as control and the other set treated with GM-CSF at 600 pg·mL^−1^. TRAP staining to compare the osteoclast differentiation in NRP2WT and NRP2KO condition in RANKL+M-CSF at **a** Day 2. **b** Day 3. in LNCAP C4-2B CM **e** Day 2 **f** day 3. Quantification and comparison of the number of TRAP-positive multinucleated osteoclast per well in NRP2WT and NRP2KO osteoclasts with and without GM-CSF in RANKL and MCSF **c** Day 2 **d** Day 3 and in LNCaP C4-2B CM **g** Day 2 **h** Day 3. All values reported as mean± SEM from two independent experiments. Statistically significant *P* value denoted as *** (0.001), **** (0.000 1), ***** (0.000 001). **i** Flow cytometric analysis of surface markers of mononucleated bone marrow cells differentiated by PC3 CM

Interestingly, GM-CSF is a potent inhibitor of early osteoclastic differentiation and fusion and is abundant in the PC3 CM (Fig. 5e, f).^41,42^ An ELISA showed low levels of GM-CSF in LNCaP C4-2B CM and a high concentration of 590 pg·mL^−1^ in PC3 CM (Fig. 5c). To test whether high GM-CSF levels can inhibit osteoclast differentiation and fusion, we treated osteoclasts with GM-CSF (600 ng·mL^−1^) combined with RANKL and M-CSF. On both days 2 and 3 of osteoclastic differentiation, GM-CSF treatment decreased the number of TRAP-positive osteoclasts and delayed their fusion (indicated by an increased number of mononuclear cells). Moreover, NRP2 deletion had no additional effect on the differentiation and fusion of these osteoclasts (Fig. 6a–d). Similar to RANKL and M-CSF treatment, GM-CSF addition to LNCaP C4-2B CM significantly reduced early differentiation of NRP2-deleted osteoclasts. Specifically, at day 2 and day 3, GM-CSF treatment decreased the total number of TRAP-positive mononuclear osteoclasts and delayed fusion of the osteoclasts following NRP2 deletion (Fig. 6e–h). These results suggest that GM-CSF is an inhibitor of early osteoclast differentiation and fusion. The GM-CSF secreted by PC3 keeps osteoclasts in a mononucleated state and thus helps the cells to overcome the regulation of NRP2.

GM-CSF in high concentrations can induce immature dendritic cell commitment, which can differentiate into TRAP-positive osteoclasts via c-Fos.^42^ Therefore, PC3 CM-induced osteoclasts can originate from immature dendritic cells rather than their osteoclastic precursors, and this lineage switch contributes to the evasion of NRP2 regulation in PC3 CM-treated osteoclasts. To examine this, mononuclear cells were cultured in PC3 CM and tested for the presence of dendritic markers. Our flow cytometric analysis revealed that these cells were CD11C positive (marker for mature dendritic cells) with intermediate expression of surface markers CD40 and CD86 and F4/80 (Fig. 6i). This result suggests that PC3 CM drives mononuclear cells to become immature dendritic cells, which then behave like osteoclasts.

### NRP2 abrogation in osteoclasts prevents PCa to grow in bone

**Fig. 7:**
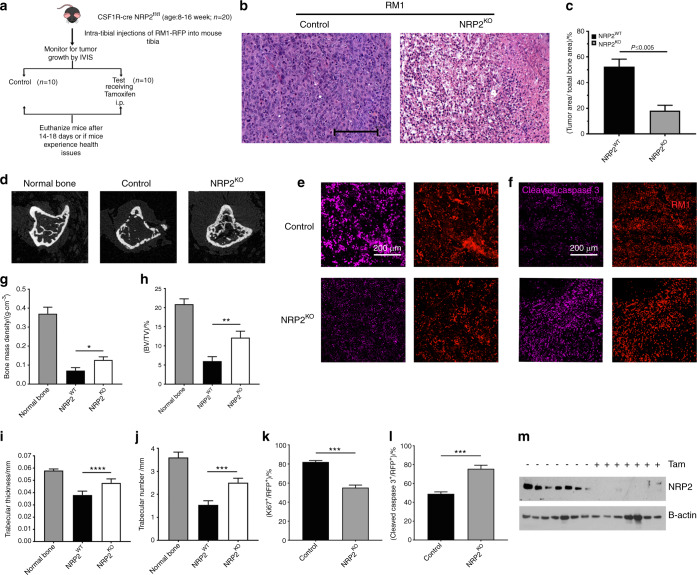
PCa-induced osteolysis is decreased by osteoclast-specific NRP2 depletion in vivo. **a** Schematic representation of the experimental design of intratibial injection of RM1 cells into transgenic compound NRP2 Fl/Fl; CSF1R-Cre mice. The mice were divided into two groups: control and test. The mice in control group received corn oil only and the test group was injected intraperitonially tamoxifen from day 3 of intratibial inoculation of RM1 cells until the end of experiment. **b** Representative H&E images of tumor growth in control and NRP2KO groups. **c** Tumor area calculated and represented as a graph with statistical significance. **d** micro-CT images of trabecular compartment of the proximal tibia showing bone changes in control and NRP2KO mice in comparison with the normal untreated bone. **g–j** Graphs showing bone mass density (BMD), bone volume/tissue volume (BV/TV), trabecular thickness and trabecular number per mm area for each group. *n* = 6 mice were used to analyze tumor bearing bones from three independent experiments. **e**, **f** Immunofluorescence images depicting the status of Ki67 and cleaved caspase 3 (pink) with respective tumor cells (red) in control and NRP2KO groups. **k**, **l** Graphical representation of the quantitation of Ki67 and cleaved caspase 3 immunoflourescence (pink) normalized with area of tumor cells (red) and statistical significance denoted as *P* value * (0.05), ** (0.01), *** (0.001). **m** Western blot depicting the osteoclastic NRP2 expression in the control and NRP2KO mice used in the experiment

PCa bone metastasis is clinically characterized by high osteoblastic and moderate osteolytic lesions. We showed that different PCa cells in bone can diversely activate osteoclasts by modulating distinct molecular mechanisms. PCa cells like LNCaP C4-2B, which predominantly form osteosclerotic lesions, activate NRP2 in osteoclasts to inhibit their function; depletion of NRP2 in LNCaP C4-2B CM-treated osteoclasts led to hyper-osteolytic function, which could prove to be fatal if NRP2 inhibition was applied as a therapy for PCa bone metastasis. Further, our cell culture system cannot recapitulate the complex communication between different cells in the bone and PCa cells, which promotes bone metastasis. To address these questions, we utilized the same transgenic mouse model that was used in the in vitro studies where NRP2 could be conditionally deleted in the myeloid population (progenitors of osteoclast) with the administration of tamoxifen (Fig. 7a). Injection of RM1 cells (mouse PCa cell line syngenic to C57BL/6 background and promotes mixed bone lesions) into the tibia of these transgenic mice significantly increased tumor growth, thereby causing severe bone loss in control mice (Fig. 7b, c). Surprisingly in the mice where the myeloid cells were depleted of NRP2, we observed decreased tumor burden in the tibia along with increased bone rescue, which contradicts our initial assumptions (Fig. 7b, c). Bone analysis showed severe bone destruction in the control group compared with the NRP2-deletion group, as confirmed by parameters such as bone mass density, bone volume/tissue volume, trabecular thickness and number (Fig. 7d, g, h, i, j). Immunofluorescence studies on the bone tissues showed that the proliferation marker Ki67 was lower in the tumor cells of the NRP2-knockout mice than in the control mice (Fig. 7e, k). Additionally, cleaved caspase 3 staining showed increased cell death in the NRP2-knockout mice compared to the control group (Fig. 7f, l). The efficacy of NRP2 knockout was confirmed by protein analysis of the osteoclasts isolated from the bone marrow of the mice from each group (Fig. 7m). These data cumulatively demonstrate that NRP2 depletion in osteoclasts rescues the bone from the deleterious effects of PCa cells by decreasing their proliferation and increasing cell death. Altogether, our study indicates that NRP2 is a promising target for treatment of PCa bone metastasis.

## Discussion

The incidence of bone metastasis in advanced-stage PCa patients is high (nearly 80%)^43^ and is one of the major reasons for patient morbidity and mortality. Treatment strategies currently used for bone metastasis are inadequate and palliative. Therefore, development of anti-metastatic agents that prevent the initiation and progression of PCa bone metastasis is crucial. The results presented here highlighted the potential of targeting the NRP2 axis as a novel therapy for PCa bone metastasis. We observed homogeneously high NRP2 expression in the majority of PCa bone metastasis specimens compared to primary PCa. Importantly, depletion of NRP2 in PCa cells when combined with chemotherapeutic stress caused tumor cell death, leading to a significant reduction of growth of metastatic PCa in bone. This result thus validated our previous in vitro studies where we observed that NRP2 deletion sensitized metastatic PCa cells to chemotherapeutic agents. Therefore, our data implicates the NRP2 axis as a potential therapeutic target for PCa bone metastasis and thus supports the future development of antibody, peptide and small molecule-based inhibitors against NRP2 as candidate drugs for bone metastatic PCa.

A systemic therapy targeting NRP2 in PCa bone metastasis is unlikely to be specific for cancer cells and could potentially block the NRP2 axis in other cells in the cancer microenvironment. Since osteoclasts express NRP2 under normal physiological condition,^20^ we evaluated whether osteoclasts differentiated by the cytokines and growth factors released by metastatic PCa cells also express NRP2 and what would be the effect of inhibiting this expression. We know that the metastatic PCa cells in bone induce osteoclasts, which are predominantly dysfunctional.^44,45^ Unlike other cancers such as breast and multiple myeloma where osteolytic lesions are predominant, the bone metastasis induced by PCa cells shows mixed osteoblastic and osteolytic lesions, with greater abundance of the former.^1,10,12,46^ Therefore, it would be difficult to predict how tumor growth in bone would be affected if a therapy perturbs the function of osteoclasts and thereby disturbs the crosstalk between tumor cells, osteoblasts and osteoclasts in the tumor microenvironment. To address this, we studied the role of NRP2 in PCa-induced osteoclasts and the effect of NRP2 inhibition on tumor growth. Our study showed that upon differentiation by PCa cells, osteoclasts express high NRP2 levels. Intriguingly, NRP2 depletion increased osteoclast differentiation and activation and increased expression of osteoclastic genes in osteoclasts treated with LNCaP C4-2B CM, which was similar to effects observed in osteoclasts treated with RANKL and M-CSF (mimicking normal physiological condition). NRP2-depleted osteoclasts formed large, multinucleated polykaryons and exhibited higher resorbing capacity than their control counterparts. Contrary to these observations, osteoclasts stimulated by osteolytic PCa cells such as PC3 did not show any change in differentiation and activation upon depletion of NRP2. Collectively, we showed that NRP2 negatively regulates the differentiation and function of osteoclasts induced by LNCaP C4-2B cells, which in turn promotes mixed lesions, as observed in the majority of PCa patients with bone metastasis. Interestingly, PC3 cells, which promote mainly osteolytic lesions, induce osteoclasts through an NRP2-independent mechanism.

The finding that NRP2 regulates osteoclastic gene expression directed our attention towards participating transcription factors. Hence, we evaluated whether NRP2 regulates the function of transcription factors NFATc1 and NF-κB, reported to be central to osteoclast differentiation.^29,47,48^ Compelling evidence from protein and immunofluorescence analysis indicated that NRP2 inhibits the expression and localization of NFATc1 when osteoclasts are induced by osteosclerotic LNCaP C4-2B or treated with RANKL and M-CSF. This suggests that NRP2 negatively regulates NFATc1, a master regulator of osteoclastogenesis. NFATc1 expression is regulated by its upstream signaling, which includes Ca^2+^ signaling.^29,49,50^ Our studies indicated that the osteoclasts devoid of NRP2 released more intracellular Ca^2+^ from ER reserves, promoted by the activation of IP3/IP3R signaling, compared with NRP2-expressing osteoclasts. We therefore concluded that the NRP2 axis regulates IP3/IP3R signaling in osteoclasts and thus controls intracellular Ca^2+^ levels and downstream activation of NFATc1. Interestingly, NRP2 also controls NFκB activation in RANKL and M-CSF-treated osteoclasts. In the absence of NRP2, NFκB works cooperatively with NFATC1 to induce osteoclast-specific genes and thus hyper-activate osteoclast function. Currently, it is unknown why NF-κB is not activated in osteoclasts induced by the CM of LNCaP C4-2B. However, this lack of NFκB activation helps to explain why the expression levels of osteoclastic genes were not comparable to the levels we observed in RANKL/M-CSF-treated osteoclasts despite significant translocation of NFATc1 in the nucleus of LNCaP C4-2B CM-treated osteoclasts following NRP2 depletion.

Although NRP2 expression was observed in PC3 CM-induced osteoclasts, NRP2 depletion did not change the differentiation and function of osteoclasts. In our initial findings, we observed that PC3 CM-induced osteoclasts expressed significant levels of osteoclastic genes and were not influenced by the status of NRP2. This hints that PC3 CM activates transcription factors that are not regulated by NRP2 to enable the expression of osteoclastic genes. We did not observe the localization of NFATc1 and NF-κB in the nucleus of PC3 CM-induced osteoclasts, suggesting that PC3 CM does not activate these transcription factors. Further studies should clarify the signaling pathways activated by PC3 CM. Also, PC3 CM induced osteoclasts in a 2–3 nucleated state, with resorptive function limited to pore formation rather than large pits. This raised the question of what in the PC3 CM is restricting the fusion and function of osteoclasts. Cytokine analysis of the PC3 CM showed high concentration of GM-CSF, which is a reported inhibitor of early differentiation and fusion in osteoclasts. Addition of GM-CSF caused a delay in the early differentiation and fusion of osteoclasts treated with RANKL and M-CSF, or LNCaP C4-2B CM. In addition, NRP2-depleted osteoclasts treated with GM-CSF also showed a lag in their differentiation and fusion. This phenomenon could be a result of uncoupling between GM-CSF and NRP2 regulation pathways, and further confirms that GM-CSF in PC3 CM restrains osteoclast differentiation and fusion. It will be important to study whether removal of GM-CSF from the PC3 CM can restore regulation by NRP2 in osteoclasts. In addition to these findings, our flow cytometric analysis of osteoclasts differentiated by PC3 CM showed characteristics of immature dendritic cells that retained the ability to differentiate into osteoclast-like cells. Furthermore, PC3 CM promoted the differentiation of mononuclear cells into immature dendritic cells, which when presented with osteoclastic growth factors became osteoclast-like cells. Thus, osteoblasts stimulated by PC3 CM evade NRP2 regulation, which is prominent in osteoclasts generated by RANKL and M-CSF and LNCaP C4-2B CM.

Our in vitro studies suggest that targeting NRP2 for treatment of bone metastasis could lead to different scenarios in PCa patients with mixed lesions. Since PCa promotes predominantly osteoblastic bone formation, an approach that enhances osteolytic activity, e.g. targeting NRP2, may interfere with prostate cancer growth in bone. To address this critical question, we used a syngenic mouse tumor model of PCa bone metastasis, where NRP2 was specifically deleted from osteoclasts. The results from these in vivo studies revealed that depletion of NRP2 from osteoclasts significantly decreases tumor burden. Even with increased osteoclasts, the bone showed less damage in the tumor-bearing NRP2-knockout mice compared to their control counterparts mainly due to less tumor growth and thus less tumor-induced osteolysis. This effect could result from two scenarios: (1) Removal of NRP2 from osteoclasts in the bone microenvironment increased osteolytic function to a level that overcame the hyper osteoblastic activity, thereby inhibiting the tumor niche in bone and inducing tumor cell death. (2) Using cre recombinase under the control of CSF1R can cause NRP2 knockout from not only osteoclasts but also cells of myeloid lineage such as macrophages and dendritic cells. Our previous work on macrophages showed that NRP2 controls their phagocytic function and its depletion can impair the clearance of apoptotic cells, leading to tumor suppression.^51^ Hence, the loss of NRP2 from macrophages may have caused a ripple effect on tumor growth inhibition in bone, which is beyond the scope of this study. Since any systemic therapy targeting NRP2 would block its function not only in osteoclasts but also in other myeloid cells, we expect our syngeneic mouse model simulates use of an NRP2 inhibitor to treat PCa bone metastasis. In summary, our study demonstrated that targeting NRP2 may prove to be effective in the treatment of PCa bone metastasis.

## Materials and methods

### Reagents

RPMI 1640 medium, DPBS, 0.25% (w/v) trypsin, MEM Non-Essential Amino Acid solution (100×), sodium pyruvate (100 mmol·L^−1^), HEPES (1 mol·L^−1^) and Penicillin-Streptomycin (5 000 U·mL^−1^) were procured from ThermoFisher Scientific. Minimum Essential Medium (MEM) Alpha Medium (10-022-CV) was purchased from Corning. Fetal bovine serum and goat serum were obtained from GIBCO.

Antibodies such as NRP2 (D39A5), NF-κB (D14E12), α-actin (D6D8), and histone H2A (D603A) were purchased from Cell Signaling Technology, NFATc1 (7A6) from ThermoFisher Scientific, HDAC1(ab7028) from Abcam, Rho-GDI (C2, sc-374579) and HSC70 (B-6, sc-7298) from Santa Cruz Biotechnology. Secondary antibodies were ordered, including goat anti-rabbit IgG-HRP (sc-2004) from Santa Cruz Biotechnology and sheep anti-mouse IgG-HRP (AC111P) from EMD Millipore. Secondary antibodies conjugated with Alexa fluor such donkey anti-rabbit Alexa Fluor 546 for NF-κB (A10040) and goat anti-mouse Alexa Fluor 660 for NFATc1 (A21054) were obtained from ThermoFisher Scientific. siRNAs against mouse NRP2 and non-targeting control (ON-TARGET plus, smart pool) were purchased from Dharmacon. Recombinant murine M-CSF (315-02) and RANK ligand (315-11) were acquired from Peprotech. TRAP staining kit was purchased from Cosmo Bio (PMC-AK04F-COS) and osteo assay surface microplates (24-well, #3987) from Corning. Reagents such as (Z)-4-Hydroxytamoxifen (H7904), HEPES, KCl, DTT, NP-40, glycerol, MgCl_2_, EDTA, PMSF, cyclosporine A, and protease inhibitors such as aprotinin and leupeptin were purchased from Sigma-Aldrich. Halt, Trizol and Powerup SYBR Green master mix were bought from ThermoFisher Scientific. The cDNA kit was obtained from Roche and primers from IDT. Fluorochrome-conjugated antibodies anti-mouse BV510 CD11c (117337), APC CD40 (124611) and PE/Cy7 CD86 (105013) were purchased from Biolegend (San Diego, CA) and anti-mouse PE F4/80 (12-4801-82) from eBioscience (San Diego, CA) for FACS. Flou-4 (F14201) was obtained from ThermoFisher Scientific.

### Mouse models

All mice used in this study were maintained under specific pathogen-free conditions. All procedures performed were in accordance with institutional guidelines and approved by the University of Nebraska Medical Center Institutional Animal Care and Use Committee (IACUC). Our study used two different mouse models, C57BL/6 and transgenic CSF1R-cre; NRP2^floxed/floxed^ mice. The C57BL/6 mice were purchased from Charles River at the age of 6–10 weeks for isolation of bone marrow.

Generation of transgenic CSF1R-cre; NRP2 Flox/Flox mice: The NRP2^floxed/floxed^ mouse was developed in Max Planck Research Unit for Neurogenetics by Dr. Peter Mombaerts. Our research collaborator in University Hospital Bonn, Germany, Dr. Michael Muders gave them as a kind gift to us. The transgenic FVB-Tg(Csf1r-Mer-iCre-Mer)1Jwp/J mice were purchased from Jackson Laboratories. These mice express a Cre recombinase/mutant murine estrogen receptor double-fusion protein under the control of the Csf1r (colony-stimulating factor 1 receptor) promoter. The Tg(Csf1r-Mer-iCre-Mer)1Jwp mice were bred with NRP2^flox/flox^ mice to obtain CSF1R-Cre; NRP2^floxed/floxed^ mice which express a tamoxifen-inducible Mer-iCre fusion protein driven by the Csf1r promoter, which upon administration with either tamoxifen intraperitoneally (75 mg·kg^−1^ body weight) in mice or (Z)-4-Hydroxytamoxifen in vitro leads to activation of Cre recombinase. The Cre recombinase targets the deletion of NRP2 in the loxP-flanked regions. The ablation of NRP2 occurs in myeloid cells as they specifically express CSF1R.

### Retrospective study of human PCa bone metastasis

A commercially available PCa bone metastases array was purchased from Tristar in 2010 (Tristar TMA number 79562475). The core diameter of each sample was 0.6 mm. The tissue microarray contained samples of fifty PCa bone metastases, five normal bones as a control, and five primary PCa as a control. A control group of primary PCa was obtained through the Biospecimen Accessioning and Processing (BAP) and Tissue and Cell Molecular Analysis (TACMA) Shared Resources at Mayo Clinic. All patients were treated between 1992 and 1997 by radical prostatectomy at Mayo Clinic under the IRB protocol. The cohort contained three groups of patients (total 130 patients). All patients had locally advanced PCa without any locoregional or distant lymph node or bone metastases (pathological stages pT3aN0 and pT3b/pT4N0). 50 patients received no adjuvant therapy, 50 patients received adjuvant hormonal therapy, and 30 patients received radiation therapy. Two board-certified pathologists scored the primary tumor and the bone metastases TMA (Raffael Jimenez and Michael Muders).

For the immunohistological staining, tissues were deparaffinized and rehydrated followed by antigen unmasking and blocking. NRP2 antibody from Santa Cruz was used (clone c9, dilution 1:150) and the cell pellet of PC3 was used as a control. As a negative control, PC3 with siRNA-mediated NRP2 depletion was used. Mouse IgG antibodies were used as an isotype control (Supplementary Fig. 1e). The Dako Envision Plus Detection Kit was used to detect staining. In the evaluation of staining, interobserver variability (Cohen’s kappa co-efficient) was tested. Based on the distribution, staining intensity or area was dichotomized ranging from none or weak, moderate to strong intensity. The differences in the staining of the primary PCa and bone metastasis was summarized using a frequency table. All tests were two-sided with *P* values less than or equal to 0.05 considered significant.

### PCa cell lines

PC3 and LNCaP C4-2B cells were cultured in RPMI complete medium supplemented with 10% fetal bovine serum (FBS) and antibiotics (Penicillin-streptomycin). RM1 cells were cultured in DMEM complete medium. Upon confluency, these cells were washed with DPBS and either briefly rinsed (LNCaP C4-2B) or treated with 0.25% (w/v) trypsin-EDTA (PC3 and RM1) to detach cells from the plate. The cells were collected in equal volumes of complete medium to neutralize the effect of trypsin. The cells were pelleted by centrifugation at 1 000 × *g* for 5 min. The cells were then suspended in fresh complete media, plated in a T-175 flask, and cultured in a tissue culture incubator maintained at 37 °C and 5% CO_2_.

### Collection of conditioned medium

To collect conditioned medium (CM) from PC3, LNCaP C4-2B and RM1, 1 × 10^6^ cells were plated in a T-75 culture flask and allowed to grow until 70% confluency. Cells were then washed with DPBS and incubated with 5 mL of fresh serum-free RPMI complete medium. The cells were then incubated for another 24 h to collect the CM. The CM was then centrifuged at 1 000 × *g* for 5 min to remove cells and cell debris and filtered using a 0.45 m filter to remove any residual debris. The CM was either used fresh for experiments or aliquoted in small volumes based on usage and stored at −80 °C for future use.

### Isolation of mouse bone marrow-derived osteoclasts

Mouse osteoclastic precursors were isolated from 6 to 10-week-old C57BL/6 male mice and transgenic mice (CSF1R-Cre; NRP2flox/flox) after euthanizing by cervical dislocation. Long bones from the mice were harvested, and the bone marrow was collected by flushing the bones with DPBS. The bone marrow was subjected to centrifugation at 2 000 × *g* for ten min, and the pellet obtained was suspended in MEM alpha medium containing 10% FBS, 1% antibiotic, 1% sodium pyruvate, and 1% MEM Non-Essential Amino Acid and HEPES (pH 7.4). The cells were then filtered through a cell strainer to remove fibrous cellular debris and make a single cell suspension. The cell suspension was then centrifuged, and the pellet was subjected to a Ficoll-Paque gradient to isolate mononuclear cells. These cells were plated in MEM alpha complete medium containing 10 ng·mL^−1^ of recombinant murine M-CSF and cultured overnight to separate macrophages (which will attach to the surface of the plate); the non-adherent cells were harvested to be used as osteoclastic precursors for all the experiments.

Three different culturing conditions were applied to mimic physiological and pathological bone metastasis. Osteoclastic precursors were differentiated into healthy osteoclasts by addition of 20 ng·mL^−1^ of recombinant M-CSF and 100 ng·mL^−1^ of recombinant RANK ligand, or CM from PC3, LNCaP C4-2B and RM1 cells (20% v/v) were mixed with MEM alpha complete medium for seven days with a boost of growth factors every alternate day from the start of the experiment. Every consecutive day, cells were supplemented with RANKL and M-CSF or CM. On day 7, the mature osteoclasts from all the three conditions were then used for isolation of protein or RNA based on the experiment conducted.

### Depletion of NRP2 using RNA interference and knockout model

NRP2 was depleted in osteoclastic precursors isolated from C57BL/6 mice using the Lonza nucleofector kit (VPA-1007) and nucleofector 2b device. The procedure followed for nucleofection was as per the manufacturer’s protocol. 25 nmol·L^−1^ of NRP2 and scrambled siRNA were used. After nucleofection, the osteoclast precursors were maintained in nucleofector solution containing medium for 8 h and then incubated with MEM alpha complete medium containing either M-CSF and RANK ligand or CM from prostate cancer cell lines and allowed to differentiate for seven days.

Osteoclastic precursors isolated from transgenic mice CSF1R-Cre; NRP2^flox/flox^ were treated with 0.3 μmol·mL^−1^ of (Z)-4-Hydroxytamoxifen to deplete NRP2 from osteoclasts specifically. A parallel set of osteoclastic precursors where no (Z)-4-Hydroxytamoxifen was added served as the control for each experiment. The control and knockout osteoclastic precursors were then differentiated into mature osteoclasts (7 days) under different conditions.

### Tartarate-resistant acid phosphatase staining

To confirm the presence of osteoclasts, TRAP staining was conducted. Cells were washed with PBS and fixed with 10% formalin at room temperature for 10 min. The osteoclasts were washed with deionized water, and 150 μL of the chromogenic substrate was added and incubated at 37 °C for 1–2 h. Cells were then washed with deionized water and 2 or more nucleated TRAP-positive cells were counted and represented graphically.

### Pit formation assay

Mouse osteoclastic precursors were plated in each well of the osteoassay plate under different conditions to analyze the efficacy of the treatment conditions. To visualize the pits, 10% bleach diluted in deionized water was added for 10 min at room temperature and washed with deionized water and dried for 3 to 5 h at room temperature. Pit clusters were observed and captured using phase contrast microscope. Images obtained were analyzed for the measurement of coated surface resorbed using ImageJ and normalized with total surface area of the well of a 24-well osteoassay plate. The percent surface area resorbed was calculated and plotted with standard error under different treatment conditions.

### Immunobloting

For protein analysis, osteoclasts were washed with PBS to remove any traces of media. The cells were lysed with ice cold lysis buffer containing CHAPS buffer pH 7.4 (40 mmol·L^−1^ HEPES, 0.3% CHAPS, 10 mmol·L^−1^ Glycerophosphate, mmol·L^−1^ sodium pyrophosphate, 2 mmol·L^−1^ EDTA) and combination of protease inhibitors, 20 µg·mL^−1^ Leupeptin, 10 µg·mL^−1^ Aprotinin, 1 mmol·L^−1^ PMSF and Halt protease.

For separation of nuclear and post-nuclear fraction in osteoclasts, 250 µL of buffer A (10 mmol·L^−1^ HEPES pH 7.8, 10 mmol·L^−1^ KCl, 2 mmol·L^−1^ MgCl_2_, 0.1 mmol·L^−1^ EDTA, 10 µg·mL^−1^ Leupeptin, 10 µg·mL^−1^ Aprotinin, 3 mmol·L^−1^ DTT, 1 mmol·L^−1^ PMSF and Halt protease) was added to each sample and incubated on ice for 17 min. 20 µL of 10% NP- 40 detergent was added to each sample containing buffer A and vortexed for 2 min and centrifuged at 14 000 r·min^−1^ for 5 min. The supernatant containing the post-nuclear proteins was separated from the pellet and labeled for protein analysis. The pellet was resuspended in 50 µL of buffer C (50 mmol·L^−1^ HEPES pH 7.8, 50 mmol·L^−1^ KCl, 300 mmol·L^−1^ NaCl, 0.1 mmol·L^−1^ EDTA, 10% (v/v) glycerol, 10 µg·mL^−1^ Leupeptin, 10 µg·mL^−1^ Aprotinin, 3 mmol·L^−1^ DTT, 1 mmol·L^−1^ PMSF and Halt protease) and vortexed and placed on a rotating rack for 1 h to dissolve the pellet containing the nuclear proteins. The dissolved pellet was then centrifuged at 14 000 r·min^−1^ for 10 min. The supernatant containing the nuclear proteins was separated from the pellet which contains DNA.

Total protein was estimated using Bradford reagent and the samples were prepared by the addition of SDS sample buffer containing β-mercaptoethanol and boiled at 95 °C for 5 min. The prepared samples were run on a precast 4%–20% Mini-PROTEAN^®^ TGX™ Gel (BioRad) and transferred on to a PVDF membrane (Life Technologies). The membrane was blocked in 5% non-fat dry milk in 1X TBST (1X Tris Buffered Saline, 0.1% Tween-20) for at least 30 min and primary antibody diluted in 1X PBS was added and incubated overnight at 4 °C with continuous shaking at low speed. On the next day, membrane was washed with 1X TBST for four times for 5 min and incubated in appropriate dilution of secondary antibody conjugated with HRP for 1 h in 1X TBST with continuous shaking at low speed at room temperature. Following this, the membranes were washed in 1X TBST every 5 min for at least 5–7 times, and the protein bands were detected using a combination dilution of SuperSignal™ West Femto Maximum Sensitivity Substrate and SuperSignal™ Pico Maximum Sensitivity Substrate captured on an X-ray film. the protein bands were identified and compared and presented as an image using ImageJ software.

### Real-time quantitative PCR

On day 7, osteoclasts were washed twice with PBS. Total RNA was isolated by adding 1 mL of TRIzol Reagent (ThermoFisher Scientific, CA) per 1 million cells as per manufacturer’s protocol and allowed to stand for 5 min at room temperature. Phase separation by chloroform followed by RNA precipitation with ethanol was done. The supernatant obtained by centrifugation was decanted, and the pellet was air-dried briefly resuspended in DEPC-treated water. The concentration and quality of the RNA were analyzed using Nanodrop Spectrophotometer.

cDNA was synthesized with Transcriptor First strand cDNA synthesis kit (Roche Diagnostics Corporation) as per the instructions provided by the manufacturers. 1 g RNA was used to generate cDNA. For real-time PCR, cDNA (50 ng) was used, and each reaction was performed in duplicates in 25 μL volume in a 96-well PCR plates using SYBR green detection system (Applied Biosystems Group) in an ABI 7500 Fast and Real-Time PCR (2 min at 50 °C, 10 min at 95 °C and 40 cycles of 15 s at 94 °C and 1 min at 60 °C) with 200–300 nmol·L^−1^ concentration of primers. The list of the primers used in this study is listed in table (Supplementary Table 1). The expression was calculated relative to that of control cells and normalized with 36B4 measured under the same conditions (Applied Biosystems/Roche, Branchburg, NJ), using the 2^–ΔΔCT^ method.

### Enzyme-linked immunosorbent assay (ELISA)

Human M-CSF and GM-CSF Quantikine ELISA kits were procured from R&D systems (cat.no. - DMC00B, DGM00, respectively) and Human RANKL from Abcam (cat.no. - ab213841) to measure the amount of these cytokines in the conditioned media obtained from PC3 and LNCaP C4-2B cell lines. The assays were conducted according to the standard protocol provided by the manufacturer. For wavelength correction, readings were obtained at 540 nm or 570 nm, and these reading were subtracted from readings at 450 to correct optical imperfections of the plate.

### Cytokine profiling assay

To detect the expression levels of cytokines and chemokines in the conditioned media, proteome profiler array (human cytokine array panel A, cat.no. ARY005) from Abcam was used. According to the array procedure, the nitrocellulose membrane containing 36 different capture antibodies was blocked with blocking buffer. The blocking buffer was removed from the membranes and the solution containing the conditioned media and the antibody cocktail was added and incubated on a rocking platform shaker overnight at 4 °C. On the following day, the membranes were washed with washed buffer thrice and 2 mL of the solution containing streptavidin-HRP was added to the membranes and incubated at room temperature for 30 min on a rocking platform shaker. The membranes were washed and chemiluminescent detection reagent mix was added sequentially developed using an x-ray film developer. The cytokines were identified by aligning the transparency overlay template on the array image and pixel density was measured using imageJ software and represented as a graph with statistical significance.

### In vivo mouse model of prostate cancer bone metastasis

All animal studies were conducted in accordance with the University of Nebraska medical center IACUC guidelines. To evaluate the role of NRP2 in PCa growing in bone and its potential in combination with chemotherapy, intratibial injections of LNCaP C4-2B were conducted in NOD SCID male mice in the age group of 8–12 weeks purchased from Jackson labs. 100 000 cells per 20 µL of LNCaP C4-2B expressing GFP- luciferase and inducible NRP2 shRNA under the control of doxycycline suspended in PBS were injected into the left tibia of mice. The right tibia received PBS only. Tumor burden in the bone was monitored by IVIS imaging and after 7 days of injection, the mice were randomized into four groups with 10 mice in each group. The first group acts as a control and received sucrose in water. The second group received doxycycline in water (2 mg·mL^−1^; 5 mg·kg^−1^ body weight) to deplete NRP2 in the cancer cells. The third group was injected intraperitoneally with docetaxel (5 mg·kg^−1^ body weight: once every 7 days for 3 weeks) and sucrose in water. The fourth group received docetaxel injection and doxycycline in water. After 3 weeks, all mice were sacrificed and the tumor cells containing bones were obtained. Bone marrow from three bones from each group were used to sort tumor cells expressing GFP by flow cytometry. These tumors cells were processed to obtain RNA and Real-time PCR was conducted to check the efficiency of NRP2 depletion. The rest of tumor bearing bones were formalin-fixed for further processing and evaluation.

Intratibial injection of RM1 expressing RFP were conducted in 8-12-week male transgenic mice (CSF1R-Cre; NRP2^floxed/floxed^) to understand the effect of NRP2 depletion in osteoclasts on the tumor growth in bone. 50 000 RM1 cells were injected into the tibia of mice and allowed to grow for four days. Since RM1 is highly osteolytic, the time frame for the tumor growth is less. On fourth day, the mice were randomized into two groups- control and test. The control group received corn oil injection and the test group received Tamoxifen (75 mg·kg^−1^ body weight from a 20 mg·mL^−1^ stock prepared in corn oil) intraperitonially (i.p.). After 14 days, the mice were euthanized and the bones with tumor cells was excised and formalin-fixed for further processing. Bone marrow from the non-tumor bearing leg was collected and processed to culture osteoclasts to check the efficiency of NRP2 depletion after administration of tamoxifen.

### Bone assessment

For micro-CT analysis, formalin-fixed bones were scanned using a high-resolution X-ray microtomography system (Skyscan 1172, Bruker, Kontich, Belgium). The X-ray source was set as follows: 55 kV and 181 μA, resolution 8.89 µm, exposure time 815 ms, and aluminum filter 0.5 mm-thick. To generate 3D images, scanning raw data were reconstructed using NRecon software. The position of each bone was corrected and saved with DataViewer. For bone quality analysis, a consistent region of interest (ROI) around tibial growth plate was identified. The ROI starts at the 50th slide above the medial tibial plateau and continues downward through tibia growth plate for 200 slides totally. The bone histomorphometric parameters, including mean bone volume (BV), bone volume/tissue volume (BV/TV), trabecular thickness (Tb.Th) and bone mineral density (BMD) were determined using CT-Analyzer software. For histomorphometry, bones were paraffin embedded, sectioned and stained for H&E. Tumor area was calculated using ventana image viewer and plotted as a bar graph.

### Immunofluorescence

Osteoclasts were grown in glass chamber slides for better attachment and convenience of staining. Osteoclasts were washed with 1X PBS three times and fixed with 4% buffered formaldehyde for 15 min. Following fixation, the cells were washed with 1X PBS and blocked with 0.3% TritonX-100 and 5% goat serum in 1X PBS for 1 h. The primary antibody (1:500 dilution) prepared in the blocking buffer was added to the cells and incubated overnight at 4 °C. On the next day, the cells washed with 1X PBS containing 0.3% TritonX-100 and incubated in the secondary antibody (Alexa-546 for NF-κB and Alexa-660 for NFATc1) diluted 1:1 000 in 0.3% TritonX-100 and 5% goat serum in 1X PBS for 1 h at 4 °C in the dark. After completion of secondary antibody incubation, the cells were washed and mounted with mounting solution containing DAPI and covered with a glass coverslip and sealed.

For bone tissues, paraffin-embedded tissues were deparaffinized and rehydrated with xylene followed by a series of ethanol washes. Antigen epitopes were revealed by boiling the tissues in 1X citrate based (pH 6.0) antigen unmasking solution (Vector laboratories) for 15 min and allowed the tissues to cool down to room temperature. The tissues were washed twice with distilled water and treated with 3% hydrogen peroxide in methanol for 20 min. Following this, the tissues were washed with distilled water and incubated in blocking buffer (5% goat serum in Tris-buffered base containing tween 20) for 1 h at room temperature. The tissues were incubated in primary antibody (1:500 dilution of Ki 67 (D3B5) and cleaved caspase 3 (Asp 175) from cell signaling) diluted in blocking buffer overnight at 4 °C. After washing with 1X PBS thrice, secondary antibody tagged with fluorochrome was applied for 1 h. The tissues were washed in 1X PBS and mounted with mounting media containing DAPI and sealed. The cells and tissues were observed using Zeiss LSM 800 with Airyscan microscope located in the UNMC confocal core facility, and data were acquired, analyzed, and processed with the Zeiss Zen 2010 software. All confocal data quantified using ImageJ software and graphical illustrations made using GraphPad Prism software.

### Intracellular Ca^2+^ Imaging

Bone marrow-derived mononuclear cells were plated on 35 mm poly-L-lysine-coated glass bottom fluorodishes (differentiated into osteoclasts in either M-CSF and RANKL or LNCaP C4-2B CM. At day 3, osteoclasts were loaded with 5 µmol·L^−1^ Flou-4 AM for 30 min at RT in loading solution: 115 mmol·L^−1^ NaCl, 5.4 mmol·L^−1^ KCl, 1 mmol·L^−1^ MgCl_2_, 20 mmol·L^−1^ Hepes, and 10 mmol·L^−1^ glucose, pH 7.4. The cells were washed with PBS thrice and loading solution with 1% FBS was added to the cells. Live cell imaging was conducted with Zeiss LSM 710 with Airyscan microscope and the fluorescent intensity was recorded simultaneously during the time series. Intracellular Ca^2+^ levels with Flou-4 AM were analyzed using excitation wavelength at 494 and an emission wavelength at 506 nm. The corrected fluorescent intensities of control and NRP2-depleted osteoclasts were calculated and represented at a graph. To inhibit the release of intracellular Ca^2+^ from the ER, 2-aminoethoxydiphenyl borate (2-APB, 50 µmol·L^−1^) was added to the cells for 30 min prior to loading Fluo-4 AM.

### Flow cytometry

Murine osteoclastic precursors were treated with PC3 CM for 24 h and then analyzed by FACS for the presence of immature dendritic markers. Briefly, after 24 h of incubation with PC3 CM, the cells were washed with PBS and incubated with fluorochrome-conjugated antibodies (BV510 CD11c, APC CD40, PE/Cy7 CD86 and PE F4/80) in FACS buffer (25 mmol·L^−1^ HEPES, 1% FBS, 1 mmol·L^−1^ EDTA in HBSS) for 30 min in dark at 4 °C. The cells were washed with PBS twice to remove unbound antibody and resuspended in FACS buffer and passed through a cell strainer and collected in FACS tubes for analysis. Data was acquired using BD LSR II with BD FACSDiva software (BD Bioscience, CA). Compensation was performed on the BD LSR II flow cytometry at the start of the experiment and data analysis was conducted on FlowJo v10 and represented as graphs.

### Statistical significance

Data are presented as mean ± standard error of the mean (SEM). Statistical analysis was performed using the standard two-tailed Student’s *t*-test using PRISM-6 software (GraphPad Inc.). Statistical comparisons of more than two groups were performed using unpaired student *t*-test. In all cases, a *P* < 0.05 was considered as highly significant.

## Supplementary information

Suplementary figure legends

Suplementary figures

Suplementary table 1

## References

[1] Macedo F (2017). Bone metastases: an overview. Oncol. Rev..

[2] Halabi S (2016). Meta-analysis evaluating the impact of site of metastasis on overall survival in men with castration-resistant prostate cancer. J. Clin. Oncol..

[3] Lund L, Borre M, Jacobsen J, Sorensen HT, Norgaard M (2008). Impact of comorbidity on survival of Danish prostate cancer patients, 1995-2006: a population-based cohort study. Urology.

[4] Terris, M. K., Qureshi, S. M. Metastatic and advanced prostate cancer. https://emedicine.medscape.com/article/454114-overview.

[5] Croucher PI, McDonald MM, Martin TJ (2016). Bone metastasis: the importance of the neighbourhood. Nat. Rev. Cancer.

[6] El-Amm J, Aragon-Ching JB (2016). Targeting bone metastases in metastatic castration-resistant prostate cancer. Clin. Med. Insights Oncol..

[7] Bienz M, Saad F (2015). Management of bone metastases in prostate cancer: a review. Curr. Opin. Support Palliat. Care.

[8] Merseburger AS (2013). Perspectives on treatment of metastatic castration-resistant prostate cancer. Oncologist.

[9] Sturge J, Caley MP, Waxman J (2011). Bone metastasis in prostate cancer: emerging therapeutic strategies. Nat. Rev. Clin. Oncol..

[10] Logothetis CJ, Lin SH (2005). Osteoblasts in prostate cancer metastasis to bone. Nat. Rev. Cancer.

[11] Chen X (2018). Osteoblast-osteoclast interactions. Connect Tissue Res.

[12] Keller ET, Brown J (2004). Prostate cancer bone metastases promote both osteolytic and osteoblastic activity. J. Cell Biochem.

[13] Guo HF, Vander Kooi CW (2015). Neuropilin functions as an essential cell surface receptor. J. Biol. Chem..

[14] Roy S (2017). Multifaceted role of neuropilins in the immune system: potential targets for immunotherapy. Front. Immunol..

[15] Yasuoka H (2009). Neuropilin-2 expression in breast cancer: correlation with lymph node metastasis, poor prognosis, and regulation of CXCR4 expression. BMC Cancer.

[16] Zhang L (2017). VEGF-A/Neuropilin 1 pathway confers cancer stemness via activating wnt/beta-catenin axis in breast cancer cells. Cell Physiol. Biochem..

[17] Zhu H, Cai H, Tang M, Tang J (2014). Neuropilin-1 is overexpressed in osteosarcoma and contributes to tumor progression and poor prognosis. Clin. Transl. Oncol..

[18] Dutta S (2016). Neuropilin-2 regulates endosome maturation and EGFR trafficking to support cancer cell pathobiology. Cancer Res..

[19] Stanton MJ (2013). Autophagy control by the VEGF-C/NRP-2 axis in cancer and its implication for treatment resistance. Cancer Res.

[20] Verlinden L (2013). Nrp2 deficiency leads to trabecular bone loss and is accompanied by enhanced osteoclast and reduced osteoblast numbers. Bone.

[21] Abida W (2019). Genomic correlates of clinical outcome in advanced prostate cancer. Proc. Natl Acad. Sci. USA.

[22] Dutta S (2016). NRP2 transcriptionally regulates its downstream effector WDFY1. Sci. Rep..

[23] Lin DL (2001). Bone metastatic LNCaP-derivative C4-2B prostate cancer cell line mineralizes in vitro. Prostate.

[24] Zhang J (2001). Osteoprotegerin inhibits prostate cancer-induced osteoclastogenesis and prevents prostate tumor growth in the bone. J. Clin. Invest..

[25] Di Giacomo V (2017). DeltaNp63alpha promotes adhesion of metastatic prostate cancer cells to the bone through regulation of CD82. Oncogene.

[26] Nandana S (2017). Bone metastasis of prostate cancer can be therapeutically targeted at the TBX2-WNT signaling axis. Cancer Res..

[27] Zhao Q, Shao J, Chen W, Li YP (2007). Osteoclast differentiation and gene regulation. Front Biosci..

[28] Kim JH, Kim N (2014). Regulation of NFATc1 in osteoclast differentiation. J. Bone Metab..

[29] Lieben L, Carmeliet G (2012). The involvement of TRP channels in bone homeostasis. Front Endocrinol. (Lausanne).

[30] Prakriya M, Lewis RS (2001). Potentiation and inhibition of Ca^2+^ release-activated Ca^2+^ channels by 2-aminoethyldiphenyl borate (2-APB) occurs independently of IP(3) receptors. J. Physiol..

[31] Feng X, Teitelbaum SL (2013). Osteoclasts: New Insights. Bone Res.

[32] Lee JH (2012). CXCL10 promotes osteolytic bone metastasis by enhancing cancer outgrowth and osteoclastogenesis. Cancer Res..

[33] Xuan W (2017). Osteoclast differentiation gene expression profiling reveals chemokine CCL4 mediates RANKL-induced osteoclast migration and invasion via PI3K pathway. Cell Biochem. Funct..

[34] Grassi F (2004). CXCL12 chemokine up-regulates bone resorption and MMP-9 release by human osteoclasts: CXCL12 levels are increased in synovial and bone tissue of rheumatoid arthritis patients. J. Cell Physiol..

[35] Rao M, Supakorndej T, Schmidt AP, Link DC (2015). Osteoclasts are dispensable for hematopoietic progenitor mobilization by granulocyte colony-stimulating factor in mice. Exp. Hematol..

[36] Li S (2015). Granulocyte colony-stimulating factor induces osteoblast inhibition by B lymphocytes and osteoclast activation by T lymphocytes during hematopoietic stem/progenitor cell mobilization. Biol. Blood Marrow. Transpl..

[37] Shiratori T (2018). IL-1beta induces pathologically activated osteoclasts bearing extremely high levels of resorbing activity: a possible pathological subpopulation of osteoclasts, accompanied by suppressed expression of Kindlin-3 and Talin-1. J. Immunol..

[38] Herrero AB (2016). Effects of IL-8 up-regulation on cell survival and osteoclastogenesis in multiple myeloma. Am. J. Pathol..

[39] Yoshitake F, Itoh S, Narita H, Ishihara K, Ebisu S (2008). Interleukin-6 directly inhibits osteoclast differentiation by suppressing receptor activator of NF-kappaB signaling pathways. J. Biol. Chem..

[40] Dai SM, Nishioka K, Yudoh K (2004). Interleukin (IL) 18 stimulates osteoclast formation through synovial T cells in rheumatoid arthritis: comparison with IL1 beta and tumour necrosis factor alpha. Ann. Rheum. Dis..

[41] Lee MS (2009). GM-CSF regulates fusion of mononuclear osteoclasts into bone-resorbing osteoclasts by activating the Ras/ERK pathway. J. Immunol..

[42] Miyamoto T (2001). Bifurcation of osteoclasts and dendritic cells from common progenitors. Blood.

[43] American Cancer Society. *Facts & Figures* (2020). 10.6004/jadpro.2020.11.2.1 PMC7848816 33532112

[44] Shiozawa Y (2011). Human prostate cancer metastases target the hematopoietic stem cell niche to establish footholds in mouse bone marrow. J. Clin. Invest.

[45] Shiozawa Y, Pienta KJ, Taichman RS (2011). Hematopoietic stem cell niche is a potential therapeutic target for bone metastatic tumors. Clin. Cancer Res.

[46] Israeli RS (2008). Managing bone loss and bone metastases in prostate cancer patients: a focus on bisphosphonate therapy. Rev. Urol..

[47] Kikuta J, Ishii M (2013). Osteoclast migration, differentiation and function: novel therapeutic targets for rheumatic diseases. Rheumatol. (Oxf.).

[48] Ikeda K, Takeshita S (2016). The role of osteoclast differentiation and function in skeletal homeostasis. J. Biochem..

[49] Novack DV, Teitelbaum SL (2008). The osteoclast: friend or foe?. Annu. Rev. Pathol..

[50] Park JH, Lee NK, Lee SY (2017). Current understanding of RANK signaling in osteoclast differentiation and maturation. Mol. Cells.

[51] Roy S (2018). Macrophage-derived neuropilin-2 exhibits novel tumor-promoting functions. Cancer Res.

