# Supplementary material for: Tumor- and osteoclast-derived NRP2 in prostate cancer bone metastases
Source: Bone Res. 2021 May 14;9:24. doi: 10.1038/s41413-021-00136-2 (PMC8121836; doi:10.1038/s41413-021-00136-2)
Supplement: Supplementary file 1 — Suplementary figure legends [file 41413_2021_136_MOESM1_ESM.docx]

**Figure 1**: Targeting NRP2 in prostate cancer cells in PCa bone metastasis is effective in combination with chemotherapy. **a** Top: Representative immunohistochemical staining of NRP2 expression in human primary prostate cancer and prostate cancer bone metastasis. Bottom: Table showing the percentage positivity of NRP2 staining intensity distinguished as No, intermediate and strong staining in human primary prostate cancer and prostate cancer bone metastasis tissues. **b** Dot-plot graph depicting the expression of NRP2 from RNA- seq data of mCRPC patients comparing between metastatic sites: bone vs other soft or visceral tissue sites. **c** Schematic diagram of the intratibial injection of LNCaP C4-2B containing inducible NRP2 shRNA. At day 7, the mice were sorted based on the bioluminescence imaging and divided into four groups: control, docetaxel alone, doxycycline in water and combination of doxycycline in water and docetaxel. **d** Representative micro-CT images of trabecular compartment of the proximal tibia for normal bone and all four treatment groups. Graphs showing bone mass density (BMD), % Bone volume/ tissue volume (BV/TV) and trabecular number per mm area for each group. For each group, N=6 mice were used to analyze tumor bearing bones from two independent experiments. **e** Representative H&E images for each treatment group depicting the status of tumor cells in the tibia of mice. **f** & **g** immunofluorescence images showing the expression of Ki67 (proliferation marker) and cleaved caspase 3 (apoptosis marker) for each group respectively in pink along with respective area showing tumor cells in green. **h** & **i** Graphs representing the quantification of the mean corrected total cell fluorescence for i67 and cleaved caspase 3 (pink) with respect to tumor cells (green). All data are shown as mean ± standard error of mean (SEM). Statistical significance was calculated by student t-test and P-value denoted as NS (not significant), * (0.05), ** (0.01), *** (0.001).

**Figure 2**: NRP2 ablation increases osteoclast differentiation. **a** Protein analysis of NRP2 expression in a time course (0-6 days) in osteoclasts differentiated under different conditions in comparison to osteoclastic precursors under the conditions of RANKL (100 ng/ml) and M-CSF (20 ng/ml), LNCaP C4-2B CM and PC3 CM. Right: Comparison of NRP2 protein levels in RANKL and M-CSF, LNCaP C4-2B CM and PC3 CM at day 6. **b** Graph showing the expression of NRP2 at mRNA level in all the three conditions in the time course of osteoclast differentiation. Knockout of NRP2 in osteoclastic precursors isolated from NRP2 ^Fl/Fl^; CSF1R-Cre transgenic mice by addition of 4-HydroxyTamoxifen *in-vitro* and differentiated into osteoclasts in the presence of RANKL and M-CSF, LNCaP C4-2B CM and PC3 CM. **c-e** TRAP staining showing osteoclast differentiation at day 4-6 after depletion of NRP2 in RANKL and M-CSF, LNCaP C4-2B CM and PC3 CM respectively. **f-h** Quantification of the TRAP positive multinucleated cells per well and comparison between NRP2^WT^ and NRP2^KO^ osteoclasts represented as a graph in RANKL and M-CSF, LNCaP C4-2B CM and PC3 CM respectively. All values reported as mean± SEM from three independent experiments. Statistically significant P-value denoted as * (0.05), ** (0.01), *** (0.001).

**Figure 3**: Depletion of NRP2 escalates the resorptive function and gene expression in osteoclasts. Knockout of NRP2 in osteoclastic precursors isolated from NRP2 ^Fl/Fl^; CSF1R-Cre transgenic mice by addition of 4-HydroxyTamoxifen *in-vitro* and differentiated into osteoclasts in the presence of RANKL and M-CSF, LNCaP C4-2B CM and PC3 CM on a 24-well osteoassay plate. **a-c** Representative images of pit resorption to compare the resorbed area in NRP2WT and NRP2KO osteoclasts in RANKL+M-CSF, LNCAP C4-2B CM and PC3 CM respectively. **d-f** Quantification and comparison of the percentage resorbed area in NRP2^WT^ and NRP2^KO^ osteoclasts in RANKL and M-CSF, LNCaP C4-2B CM and PC3 CM respectively. **g-i** Graphical representation of the osteoclastic gene expression at mRNA in NRP2^WT^ and NRP2^KO^ condition treated respectively with RANKL and M-CSF, LNCaP C4-2B CM and PC3 CM. All values reported as mean± SEM from three independent experiments and represented as a bar graph with error bar. Statistically significant P-value denoted as * (0.05), ** (0.01), *** (0.001).

**Figure 4**: NFATc1 is regulated by NRP2. NRP2 depletion in osteoclastic precursors isolated from NRP2 ^Fl/Fl^; CSF1R-Cre transgenic mice by addition of 4-HydroxyTamoxifen *in-vitro* and differentiated into osteoclasts under different conditions. Confocal images showing NFATc1 translocation into the nucleus compared between NRP2^WT^ and NRP2^KO^ osteoclasts at day 3 of osteoclastic differentiation in **a** RANKL and M-CSF, **e** LNCaP C4-2B CM. Insert represents the total field from which the magnified image was taken. **b, f.** Quantification of the mean corrected total cell fluorescence between NRP2^WT^ and NRP2^KO^ osteoclasts in RANKL and M-CSF and LNCaP C4-2B CM respectively. All values reported as mean± SEM from three independent experiments and represented as a bar graph with error bar. The statistical significance, P-value depicted as *(<0.05),**(<0.01), ***(<0.001). **c, g** Western blot showing the total NFATc1 protein in NRP2^WT^ and NRP2^KO^ osteoclasts at day 3 in RANKL and M-CSF as well as LNCaP C4-2B CM respectively. Mean Fluorescence Intensity proportional to the levels of intracellular Ca^2+^ in NRP2^WT^ and NRP2^KO^ osteoclasts at day 3 of osteoclastic differentiation with the addition of 2-APB, an allosteric inhibitor of IP3-induced Ca^2+^ release in **d** RANKL and M-CSF, **h** LNCaP C4-2B CM. Western blot analysis of NF-κB translocation status in nuclear and post nuclear fractions of protein lysates isolated from osteoclasts depleted of NRP2 under the conditions of **i** RANKL and M-CSFand **j** LNCaP C4-2B CM. Rho-GDI and HDAC1 used as loading controls for post-nuclear and nuclear proteins respectively. **k** Schematic illustration of the molecular pathways through which NRP2 regulates the gene transcription in osteoclasts. In RANKL and M-CSF, NRP2 inhibits NFATc1 and NF-κB translocation into the nucleus while only NFATc1 nuclear translocation is blocked by NRP2 in LNCaP C4-2B CM via the regulation of Ca^2+^ release from endoplasmic reticulum.

**Figure 5**: Cytokine analysis of CM collected from LNCaP C4-2B and PC3. Graphical representation of absolute **a** RANKL, **b** M-CSF and **c** GM-CSF concentration in pg/ml in LNCaP C4-2B and PC3 CM by ELISA. **d** Osteoclastic precursors in NRP2^WT^ and NRP2^KO^ condition treated with PC3 CM without and with RANKL at 50 ng/ml and graphically represented in **g**. **e** Human cytokine array membrane containing 36 different cytokines were incubated with CM from LNCaP C4-2B and PC3 mixed with biotynylated detection antibodies. The bound cytokine developed with Streptavidin-HRP and chemiluminescent detection reagents. Light produced at each spot is proportional to the amount of cytokine bound. Dot blot showing the presence of cytokine in LNCaP C4-2B CM (left) and PC3 CM (right) along with the table showing the complete list of cytokines that were found in the CM. **f** Average pixel density of each cytokine observed in the dot blots was analyzed by Imagej software and graphically represented to compare the cytokines obtained in LNCaP C4-2B and PC3 CM.

**Figure 6:** Addition of GM-CSF blocks the differentiation and fusion of osteoclasts. Knockout of NRP2 in osteoclastic precursors isolated from NRP2 Fl/Fl; CSF1R-Cre transgenic mice by addition of 4-HydroxyTamoxifen in-vitro and differentiated into osteoclasts in the presence of RANKL and M-CSF, LNCaP C4-2B CM and PC3 CM. Under these conditions, one set as control and the other set treated with GM-CSF at 600 pg/ml. TRAP staining to compare the osteoclast differentiation in NRP2^WT^ and NRP2^KO^ condition in RANKL+M-CSF at **a** Day 2. **b** Day 3. in LNCAP C4-2B CM **e** Day 2 **f** day 3. Quantification and comparison of the number of TRAP-positive multi-nucleated osteoclast per well in NRP2^WT^ and NRP2^KO^ osteoclasts with and without GM-CSF in RANKL and M-CSF **c** Day 2 **d** Day 3 and in LNCaP C4-2B CM **g** Day 2 **h** Day 3. All values reported as mean± SEM from two independent experiments. Statistically significant P-value denoted as *** (0.001), **** (0.0001), ***** (0.000001). **i** Flow cytometric analysis of surface markers of mononucleated bone marrow cells differentiated by PC3 CM.

**Figure 7:** PCa-induced osteolysis is decreased by osteoclast-specific NRP2 depletion *in-vivo*. **a** Schematic representation of the experimental design of intratibial injection of RM1 cells into transgenic compound NRP2 Fl/Fl; CSF1R-Cre mice. The mice were divided into two groups: control and test. The mice in control group received corn oil only and the test group was injected intraperitonially tamoxifen from day 3 of intratibial inoculation of RM1 cells until the end of experiment. **b** Representative H&E images of tumor growth in control and NRP2^KO^ groups. **c** Tumor area calculated and represented as a graph with statistical significance. **d** micro-CT images of trabecular compartment of the proximal tibia showing bone changes in control and NRP2^KO^ mice in comparison with the normal untreated bone. **g- j** Graphs showing bone mass density (BMD), % Bone volume/ tissue volume (BV/TV), trabecular thickness and trabecular number per mm area for each group. N=6 mice were used to analyze tumor bearing bones from three independent experiments. **e, f** Immunofluorescence images depicting the status of Ki67 and cleaved caspase 3 (pink) with respective tumor cells (red) in control and NRP2^KO^ groups. **k, l** Graphical representation of the quantitation of Ki67 and cleaved caspase 3 immunoflourescence (pink) normalized with area of tumor cells (red) and statistical significance denoted as p-value * (0.05), ** (0.01), *** (0.001). **m** Western blot depicting the osteoclastic NRP2 expression in the control and NRP2^KO^ mice used in the experiment.

**Supplementary Figure 1: a** Immunohistochemical staining of NRP2 in PC3 cells treated with either scrambled siRNA or NRP2 siRNA to confirm the specificity of the NRP2 antibody used for staining. **b** Graphical representation of NRP2 expression at mRNA level relative to 36B4 in LNCaP C4-2B cells sorted from the tumor bearing bones from intratibial injection of LNCaP C4-2B cells in SCID mice. Three mice each from the four groups of the treatment- control, docetaxel only, NRP2 shRNA and NRP2 shRNA combined with docetaxel were used from each experiment and a total of six mice from two independent experiments were used to confirm the depletion of NRP2 from LNCaP C4-2B. Data was statistically quantitated and p-value depicted as NS- not significant, *(<0.05),**(<0.01), ***(<0.001), **** (<0.0001). **c** Graphical representation of the weight of mice measured in grams at different days for each group in the experiment with intrabial injection of LNCaP C4-2B cells in mice. **d** Graph depicting the water consumption containing either sucrose only or doxycycline in sucrose water for the respective experimental group for the intratibial inoculation of LNCaP C4-2B cells in mice. The p-value in both the graphs is represented as NS- not significant. **e** Immunohistochemical staining of NRP2 in human prostate with normal prostatic glands as high grade prostatic intraepithelial neoplasia.

**Supplementary Figure 2:** Osteoclasts differentiated from bone marrow derived osteoclastic precursors during day 4, 5 and 6 of differentiation under the conditions of RANKL and M-CSF, LNCaP C4-2B CM and PC3 CM. Representative images of **a, c, e** TRAP staining and **b, d, f** pit resorption is depicted for each condition for day 4, 5 and 6. **g** graphical representation of the osteoclastic genes’ mRNA expression in each condition calculated relative to the housekeeping gene 36B4. **h** Graph showing the TRAP activity of the osteoclasts differentiated under the three conditions of treatment.

**Supplementary Figure 3:** Depletion of NRP2 from osteoclastic precursors isolated from the NRP2 ^Fl/Fl^; CSF1R-Cre transgenic mice by addition of 4-HydroxyTamoxifen increases TRAP activity. At day 6 of differentiation, TRAP activity was measured at 540 nm and represented as a graph comparing the NRP2^WT^ and NRP2^KO^ osteoclasts treated with **a** RANKL and M-CSF, **c** LNCaP C4-2B CM and **e** PC3 CM.  **b, d, f** Western blot showing depletion of NRP2 at day 4-6 in RANKL and M-CSF, LNCaP C4-2B CM and PC3 CM respectively.

**Supplementary Figure 5:** NRP2 depletion causes an increase in osteoclast differentiation and gene expression. NRP2 was depleted in osteoclastic precusors isolated from C57B/L6 mice with NRP2 siRNA by nucleoporation and treated with RANKL and M-CSF, LNCaP C4-2B CM and PC3 CM simultaneously to differentiate into osteoclasts. Scrambled siRNA (Scr) was used as a control. TRAP staining and graphical depiction of TRAP activity for **a, e** RANKL and M-CSF, **b, g** LNCaP C4-2B CM and **c, i** PC3 CM are shown. **d, f, h** Graph showing the efficiency of siRNA to deplete NRP2 in each condition. **j, k, l** graphical representation of osteoclastic gene expression profile comparing osteoclasts treated with scrambled siRNA and NRP2 siRNA for each of the three conditions. All data has been repeated twice to attain statistical significance and p-value denoted as * (0.05), ** (0.01), *** (0.001).

**Supplementary Figure 5:** NRP2 regulates the functions of NFATC1. NRP2 depletion in osteoclastic precursors isolated from NRP2 ^Fl/Fl^; CSF1R-Cre transgenic mice by addition of 4-HydroxyTamoxifen *in-vitro* and differentiated into osteoclasts under different conditions. Confocal images showing NFATc1 translocation into the nucleus compared between NRP2^WT^ and NRP2^KO^ osteoclasts at day 2 of osteoclastic differentiation in **a** RANKL and M-CSF, **b** LNCaP C4-2B CM. Insert represents the total field from which the magnified image was taken. **c, e.** Quantification of the mean corrected total cell fluorescence between NRP2^WT^ and NRP2^KO^ osteoclasts in RANKL and M-CSF and LNCaP C4-2B CM respectively. All values reported as mean± SEM from three independent experiments and represented as a bar graph with error bar. The statistical significance, P-value depicted as *(<0.05),**(<0.01), ***(<0.001). **d** Western blot showing the total NFATc1 protein in NRP2^WT^ and NRP2^KO^ osteoclasts at day 2 in RANKL and M-CSF as well as LNCaP C4-2B CM respectively.

**Supplementary Figure 6:** Osteoclasts induced by PC3 CM evades the inhibition of NRP2. Osteoclastic precursors depleted of NRP2 by 4-HydroxyTamoxifen and differentiated in the presence of PC3 CM. **a, b** Immunofluorescence images showing the status of NFATc1 in NRP2^WT^ and NRP2^KO^ osteoclasts induced by PC3 CM at day 2 and day 3 of differentiation. Inset is the total field from which the magnified image was taken. **c** protein analysis of NF-kB in osteoclasts induced by PC3 CM in post-nuclear and nuclear fractions of the cell lysate isolated from NRP2^WT^ and NRP2^KO^ osteoclasts. Rho-GDI and HDAC1 used as loading standards for membrane and nuclear proteins.

**Supplementary Figure 7:** NRP2 depletion causes increased translocation of NFkB into the nucleus. **a** Confocal images showing NF-κB translocation into the nucleus compared between NRP2WT and NRP2KO osteoclasts at day 3 of osteoclastic differentiation in RANKL and M-CSF. **b** graph represents the quantification of the mean corrected total cell fluorescence between NRP2^WT^ and NRP2^KO^ osteoclasts in RANKL and M-CSF.
