# Supplementary material for: Tumor- and osteoclast-derived NRP2 in prostate cancer bone metastases
Source: Bone Res. 2021 May 14;9:24. doi: 10.1038/s41413-021-00136-2 (PMC8121836; doi:10.1038/s41413-021-00136-2)
Supplement: Supplementary file 2 — Suplementary figures [file 41413_2021_136_MOESM2_ESM.pdf]

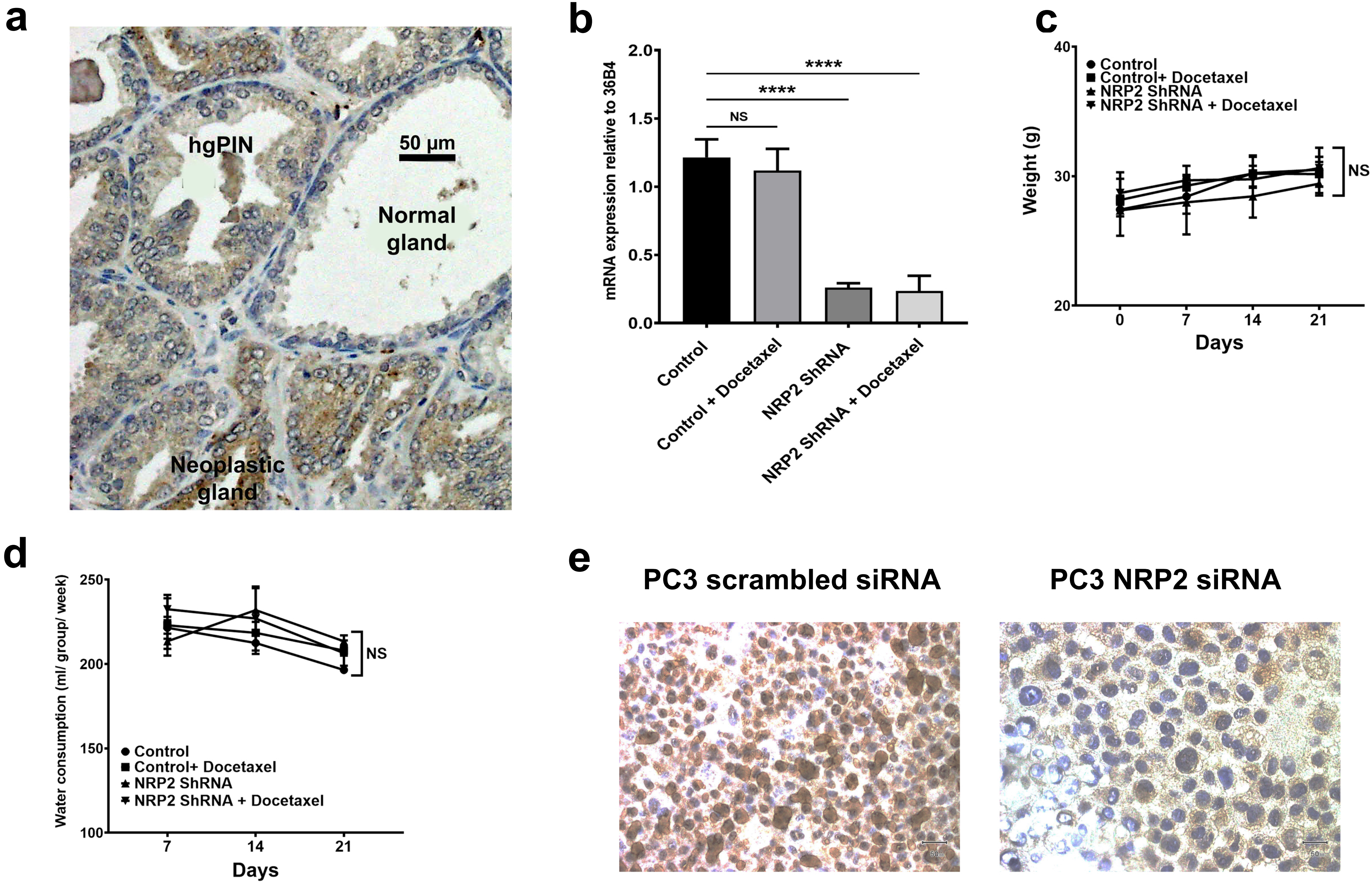
a
hgPIN
50 µm
Normal gland
Neoplastic gland
b
mRNA expression relative to 36B4
2.0
1.5
1.0
0.5
0.0
****
****
NS
Control
Control + Docetaxel
NRP2 ShRNA
NRP2 ShRNA + Docetaxel
c
Weight (g)
40
30
20
0
7
14
21
Days
Control
Control+ Docetaxel
NRP2 ShRNA
NRP2 ShRNA + Docetaxel
NS
d
Water consumption (ml/ group/ week)
250
200
150
100
7
14
21
Days
NS
Control
Control+ Docetaxel
NRP2 ShRNA
NRP2 ShRNA + Docetaxel
e
PC3 scrambled siRNA
PC3 NRP2 siRNA

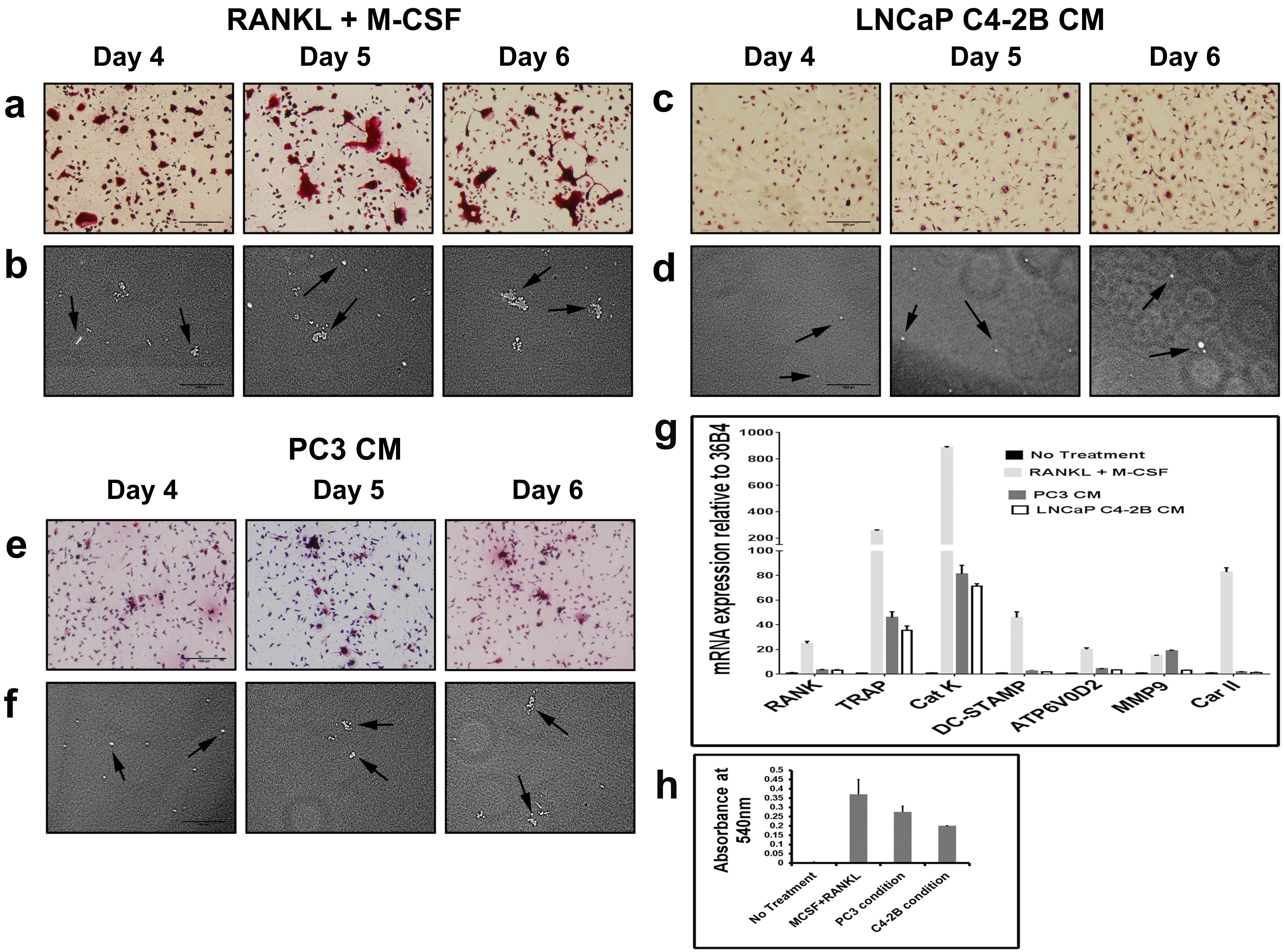
RANKL + M-CSF
Day 4
Day 5
Day 6
a
b
LNCaP C4-2B CM
Day 4
Day 5
Day 6
c
d
PC3 CM
Day 4
Day 5
Day 6
e
f
g
mRNA expression relative to 36B4
No Treatment
RANKL + M-CSF
PC3 CM
LNCaP C4-2B CM
RANK
TRAP
Cat K
DC-STAMP
ATP6V0D2
MMP9
Car II
h
Absorbance at 540nm
No Treatment
MCSF+RANKL
PC3 condition
C4-2B condition

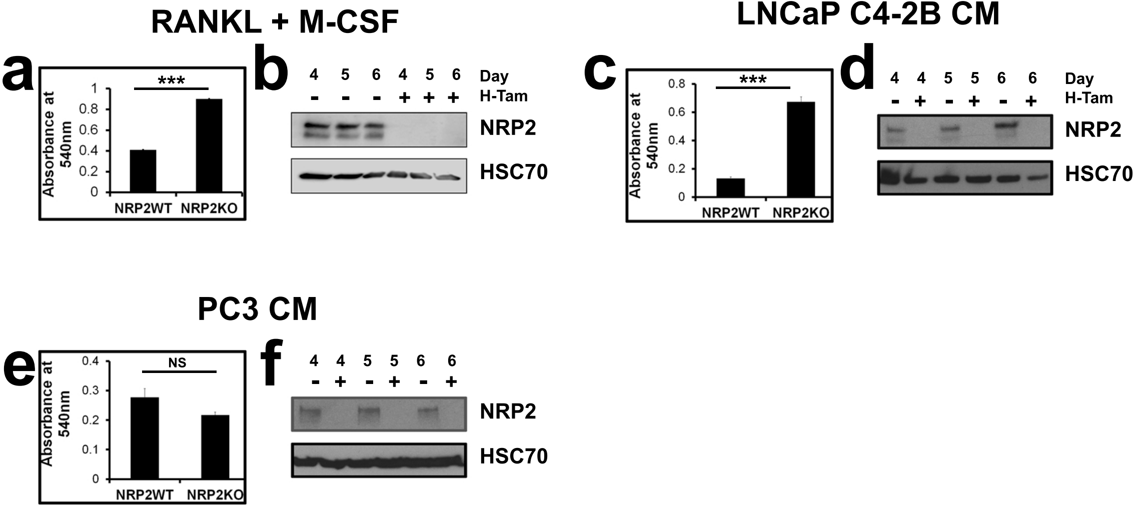

RANKL + M-CSF
a
***
Absorbance at 540nm
1
0.8
0.6
0.4
0.2
0
NRP2WT
NRP2KO
b
4 5 6 4 5 6 Day
- - - + + + H-Tam
NRP2
HSC70
LNCaP C4-2B CM
c
***
Absorbance at 540nm
0.8
0.6
0.4
0.2
0
NRP2WT
NRP2KO
d
4 4 5 5 6 6 Day
- + - + - + H-Tam
NRP2
HSC70
PC3 CM
e
NS
Absorbance at 540nm
0.4
0.3
0.2
0.1
0
NRP2WT
NRP2KO
f
4 4 5 5 6 6
- + - + - +
NRP2
HSC70

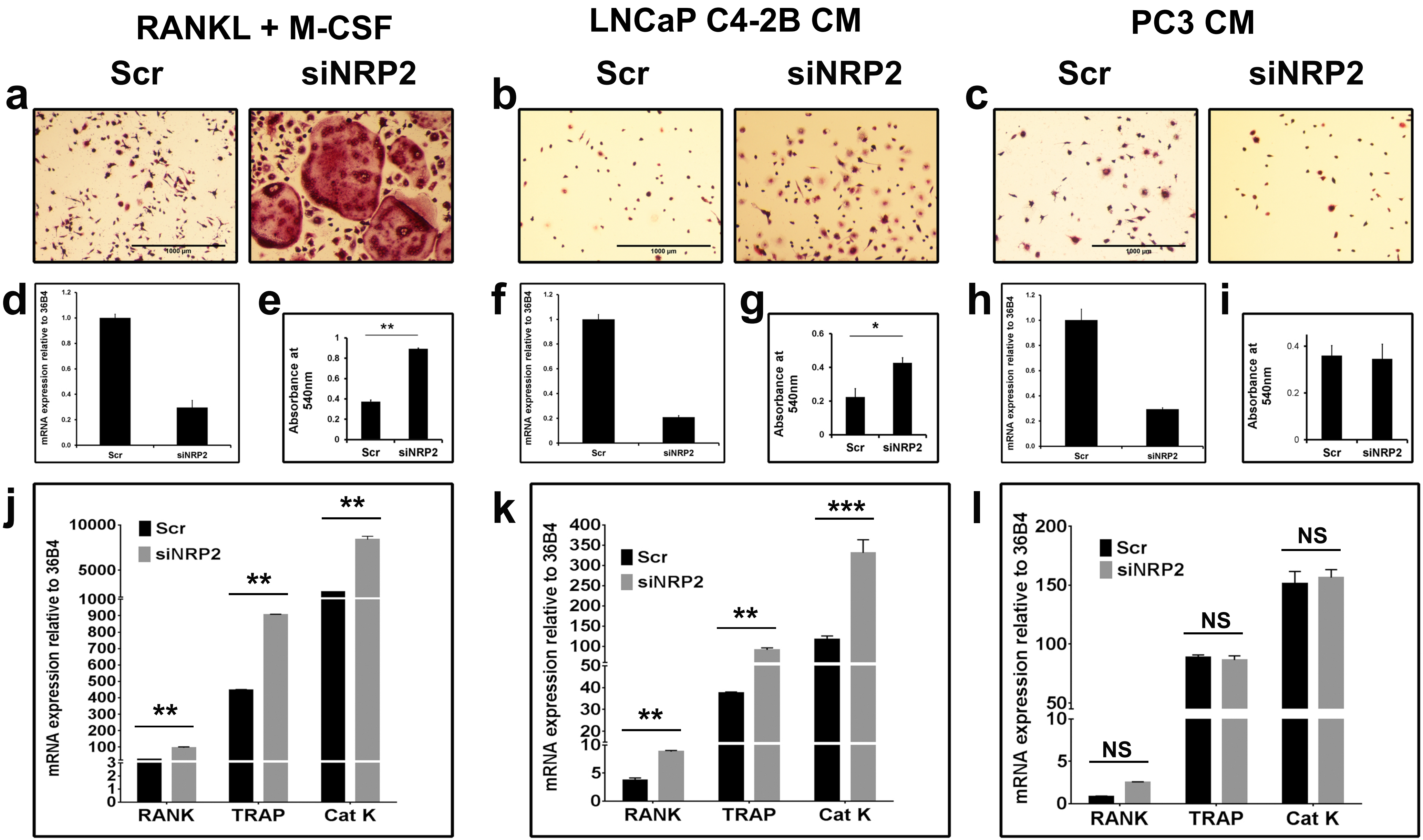

RANKL + M-CSF
LNCaP C4-2B CM
PC3 CM
Scr
siNRP2
a
b
c
1000 µm
d
e
f
g
h
i
mRNA expression relative to 36B4
Absorbance at 540nm
**
*
Scr
siNRP2
j
k
l
RANK
TRAP
Cat K
***
NS

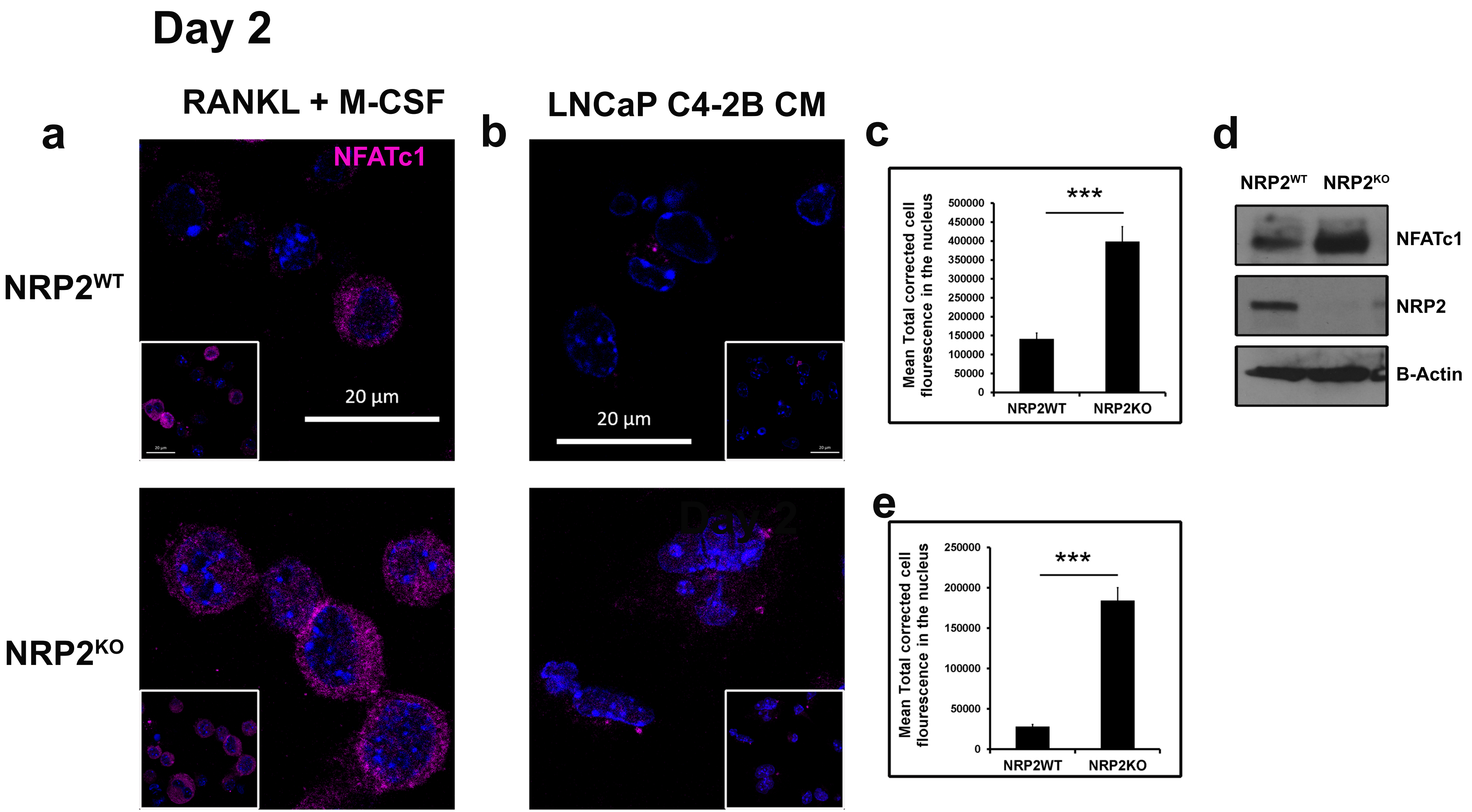

Day 2
RANKL + M-CSF
LNCaP C4-2B CM
a
b
c
d
e
NFATc1
NRP2WT
NRP2KO
20 µm
20 µm
Mean Total corrected cell flourescence in the nucleus
500000
450000
400000
350000
300000
250000
200000
150000
100000
50000
0
***
NRP2WT
NRP2KO
NRP2WT
NRP2KO
NFATc1
NRP2
B-Actin
Mean Total corrected cell flourescence in the nucleus
250000
200000
150000
100000
50000
0
***
NRP2WT
NRP2KO

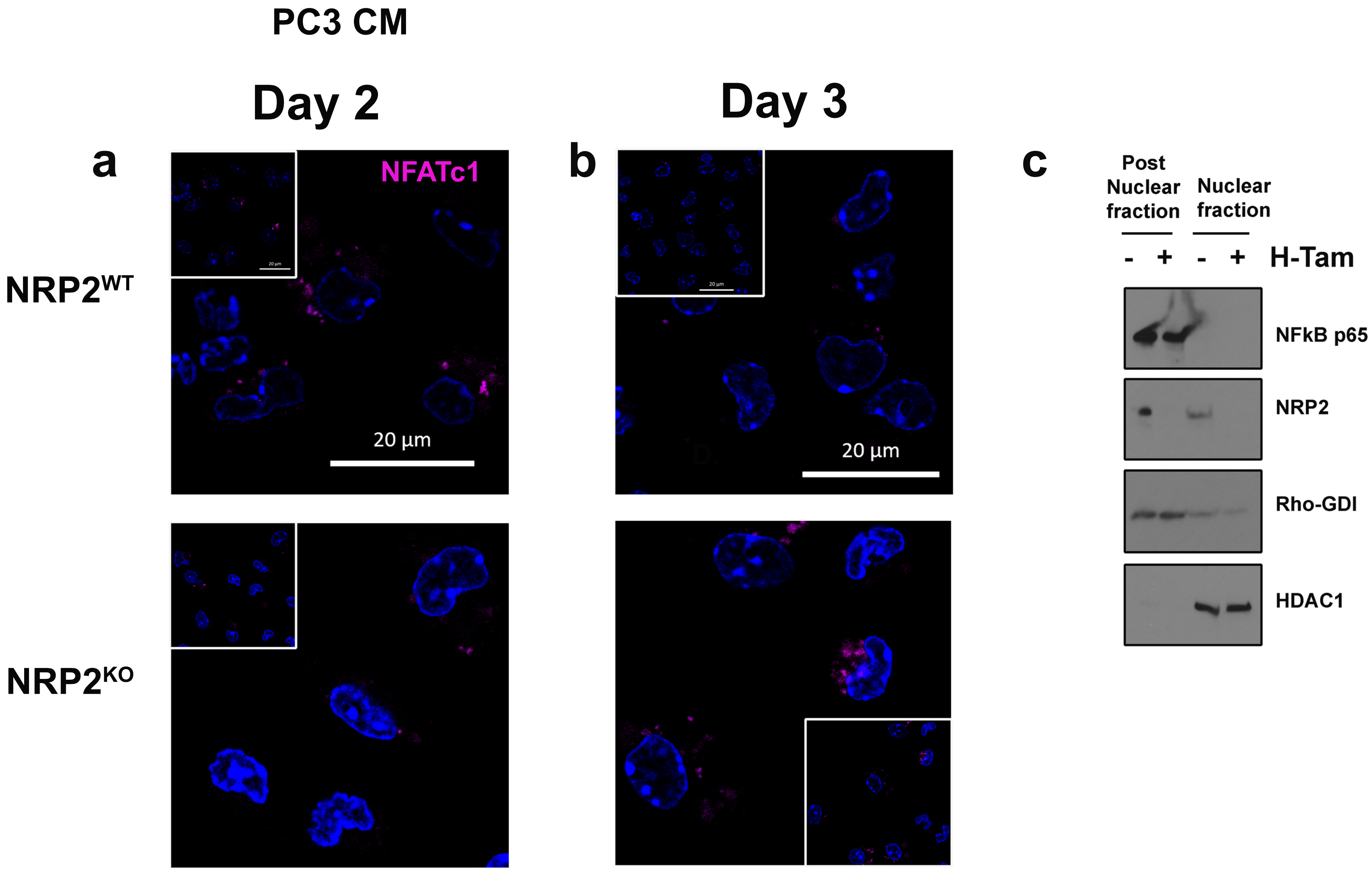
PC3 CM
Day 2
Day 3
a
b
c
NFATc1
NRP2WT
NRP2KO
20 µm
20 µm
Post Nuclear fraction
Nuclear fraction
- + - + H-Tam
NFkB p65
NRP2
Rho-GDI
HDAC1

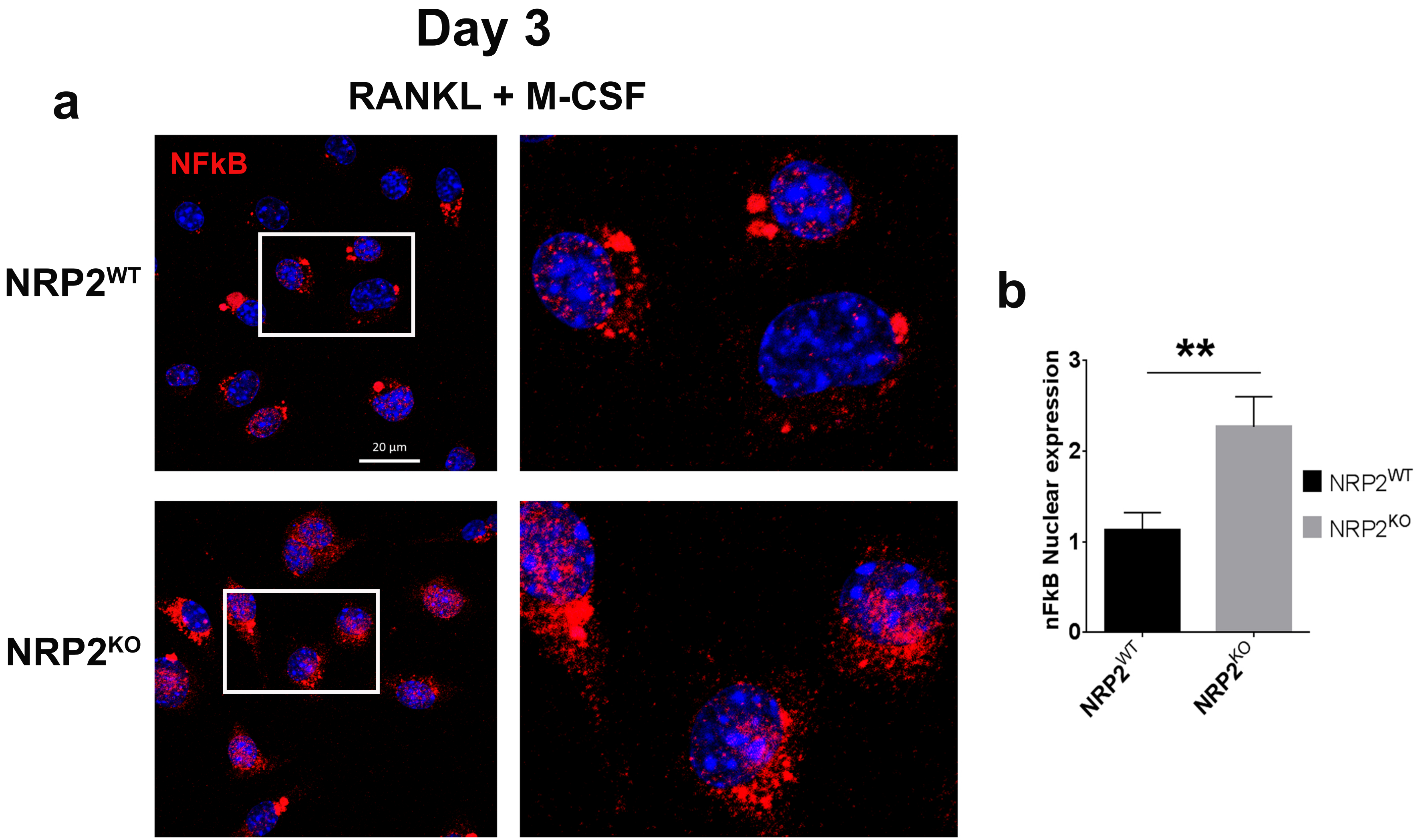
Day 3
a
RANKL + M-CSF
NFkB
NRP2WT
NRP2KO
20 µm
b
nFkB Nuclear expression
**
0
1
2
3
NRP2WT
NRP2KO
NRP2WT
NRP2KO
