# Supplementary material for: Tumor- and osteoclast-derived NRP2 in prostate cancer bone metastases
Source: Bone Res. 2021 May 14;9:24. doi: 10.1038/s41413-021-00136-2 (PMC8121836; doi:10.1038/s41413-021-00136-2)
Supplement: Supplementary file 3 — Suplementary table 1 [file 41413_2021_136_MOESM3_ESM.pdf]

Gene Name	Primer sequence
36B4	FW: ATGCAGCAGATCCGCATGT<br>RV: TCATGGTGTTCTTGCCCATCA
NRP2	FW: GTGAAGAGTGAAGAGACTACCA<br>RV: GCTGAAGTTTTCCCCACACT
TRAP	FW: TACTTCACTGGAGTGCACGAT<br>RV: GAAGTTCCAGCGCTTGGAGA
Cathepsin K	FW: GAGGGCCAACTCAAGAAGAA<br>RV: GCCGTGGCGTTATACATACA
DC-STAMP	FW: GTATCGGCTCATCTCCTCCA<br>RV: ACTCCTTGGGTTCCTTGCTT
ATP6DOV2	FW: AGAGGGGTTGCGGTTGTTAG<br>RV: GCCAGTGAGCAGGAAGTCAT
MMP9	FW: CTTAGATCATTCCAGCGT<br>RV: CAGATACTGGATGCCGTCTA
Carbonic anhydrase II	FW: ATTGGACCTGCCTCACAAGG<br>RV:CCACATGAGACACCTGGGTC
VEGF-C	FW: CAGTTGCGGTCTGTGTCCAGCGTAG<br>RV: GGACACACATGGAGGTTTAAAGAAG
RANK	FW: CAAACCTTGGACCAACTGCAC<br>RV: TGGTCTCCTCAGTGTCATGGAAG

Supplementary Table 1: Table showing the mouse-specific forward and reverse primers used in the gene expression analysis by real-time PCR.
